# NAT10‐mediated mRNA N4‐acetylcytidine modification promotes bladder cancer progression

**DOI:** 10.1002/ctm2.738

**Published:** 2022-05-06

**Authors:** Ganping Wang, Ming Zhang, Yiming Zhang, Yanqi Xie, Jiepeng Zou, Jianye Zhong, Zhijia Zheng, Xianghui Zhou, Yuhang Zheng, Binshen Chen, Chunxiao Liu

**Affiliations:** ^1^ Department of Urology Zhujiang Hospital, Southern Medical University Guangzhou China; ^2^ Department of Oral and Maxillofacial Surgery, Hospital of Stomatology, Guanghua School of Stomatology Sun Yat‐sen University Guangzhou China; ^3^ Department of Urology, The Second Affiliated Hospital, School of Medicine Zhejiang University Hangzhou China

**Keywords:** bladder cancer, mRNA, N4‐acetylcytidine, NAT10

## Abstract

**Background:**

Dysregulation of the epitranscriptome causes abnormal expression of oncogenes in the tumorigenic process. Previous studies have shown that NAT10 can regulate mRNA translation efficiency through RNA acetylation. However, the role of NAT10‐mediated acetylation modification in bladder cancer remains elusive.

**Methods:**

The clinical value of NAT10 was estimated according to NAT10 expression pattern based on TCGA data set and the tumor tissue array. Acetylated RNA immunoprecipitation sequencing was utilized to explore the role of NAT10 in mRNA ac4C modification. Translation efficiency and mRNA stability assay were applied to study the effect of NAT10‐deletion on target genes. The nude mouse model and genetically engineered mice were conducted to further verify the characteristics of NAT10 in promoting BLCA progression and regulating downstream targets.

**Results:**

NAT10 was essential for the proliferation, migration, invasion, survival and the stem‐cell‐like properties of bladder cancer cell lines. NAT10 was responsible for mRNA ac4C modification in BLCA cells, including BCL9L, SOX4 and AKT1. Deficient NAT10 in both xenograft and transgenic mouse models of bladder cancer reduced the tumor burden. Furthermore, acetylated RNA immunoprecipitation sequencing data and RNA immunoprecipitation qPCR results revealed that NAT10 is responsible for a set of ac4C mRNA modifications in bladder cancer cells. Inhibition of NAT10 led to a loss of ac4C peaks in these transcripts and represses the mRNA's stability and protein expression. Mechanistically, the ac4C reduction modification in specific regions of mRNAs resulting from NAT10 downregulation impaired the translation efficiency of BCL9L, SOX4 and AKT1 as well as the stability of BCL9L, SOX4.

**Conclusions:**

In summary, these findings provide new insights into the dynamic characteristics of mRNA's post‐transcriptional modification via NAT10‐dependent acetylation and predict a role for NAT10 as a therapeutic target in bladder cancer.

**Highlights:**

1. NAT10 is highly expressed in BLCA patients and its abnormal level predicts bladder cancer progression and low overall survival rate. NAT10 is necessary and sufficient for BLCA tumourigenic properties.
2. NAT10 is responsible for ac4C modification of target transcripts, including BCL9L, SOX4 and AKT1.
3. NAT10 may serve as an effective and novel therapeutic target for BLCA.

## Graphical abstract

1. NAT10 is highly expressed in BLCA patients and its abnormal level predicts bladder cancer progression and low overall survival rate. NAT10 is necessary and sufficient for BLCA tumorigenic properties.
2. NAT10 is responsible for ac4C modification of target transcripts, including BCL9L, SOX4 and AKT1.
3. NAT10 may serve as an effective and novel therapeutic target for BLCA.

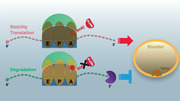

## 1 INTRODUCTION

Bladder urothelial carcinoma (BLCA) is one of the most common malignancies in the urinary system. Approximately 550 000 people are diagnosed with bladder cancer each year worldwide while the ratio of men to women is approximately 3.3: 1.^1^ According to the cancer annual report issued by the China Anti‐Cancer Center, the incidence of bladder cancer has increased over the years. In 2014, the number of new bladder cancer cases in China reached 78 100, accounting for 2.05% of all malignant tumours.^2^ Tumour recurrence after complete resection and advanced tumours remains a daunting challenge in BLCA treatment.^3^ Therefore, the identification of specific biological markers for screening bladder cancer as well as the research of effective targets for molecular therapy has become a major issue to be addressed.

Abnormal regulation of RNA epigenetics plays a key role in affecting mRNA stability and translation efficiency in tumours.^4^ The process of epigenetic modification of mRNA is reversible.^5^ The level of m^6^A, m^7^G or ac4C modifications in mRNA‐specific motifs is variable depending on protein‐binding, resulting in changes to mRNA stability and translation efficiency.^6^, ^7^, ^8^, ^9^, ^10^, ^11^ These modifications are responsible for tumour proliferation, metastasis, maintenance of stemness and cell‐fate decisions.^10^, ^12^, ^13^, ^14^ Previous studies demonstrate that METTL3, an N6‐methyltransferase that methylates adenosine residues at the N6 position of some RNAs, is involved in the m^6^A modification of ITGA6 and the AFF4/NF‐κB/MYC pathway.^15^, ^16^ However, the particular mechanism of epigenetic modification in regulating BLCA progression remains largely unknown, highlighting the quest for further research.

N4‐acetylcytidine (ac4C) is a highly conserved modification of RNA.^17^ N‐acetyltransferase 10 (NAT10), a catalytic enzyme involved in the acetylation modification of tRNA, rRNA and mRNA, has been proven to be integrated with a variety of tumours and Hutchinson‐Gilford syndrome.^10^, ^18^, ^19^, ^20^, ^21^, ^22^, ^23^, ^24^ With the catalysis of NAT10, ac4C modification on mRNA occurs at the wobble cytidine site, thereby improving mRNA stability and translation efficiency.^10^ NAT10 mediates the ac4C modification at positions 1842 and 1337 of 18S rRNA in mammals.^20^ NAT10 also participates in modifying different types of RNA in the nucleolus, including the splicing of precursor rRNA at positions A0, A1 and A2 in yeast.^22^ According to the most recent report, the high expression of the mRNA ac4C writer NAT10 indicates poorer prognosis of various malignancies and NAT10‐mediated ac4C modification on mRNA promotes gastric cancer metastasis and EMT.^25^ Although NAT10‐mediated modification of ac4C peaks on RNA and proteins has been elucidated, knowledge about the role of ac4C on mRNA and its regulatory networks in cancer remains intricate to this day. Here, we found that NAT10 directly regulates the transcripts of BCL9L, SOX4 and AKT1, increasing their stability and translation efficiency by acetylation at the wobble site. Recent studies verify a role for BCL9/9L as a supporter of tumours driven by acute APC loss, advancing colonic oncogenesis by activating Wnt signaling transduction.^26^ In addition, reports affirm that SOX4 plays an essential function in various kinds of malignancies including the leukemic phenotype of C/EBPa mutant AML and pancreatic ductal adenocarcinoma driven by cooperation of Klf5 and SOX4.^27^, ^28^ Moreover, AKT1 is a member of the AKT family, serving as an element of PIPP regulated AKT1‐dependent breast cancer growth and metastasis.^29^ Coincidently, in terms of antiviral defence and antitumour immunity, AKT1 is recruited by HER2 to block STING signalling, subsequently downregulating IFN expression.^30^

Chemically induced bladder cancer in *C57BL/6J* genetic background mice are ideal animal models for basic BLCA research. Drinking water supplemented with N‐Butyl‐N‐(4‐hydroxybutyl) nitrosamine (BBN) given to mice has been reported to construct a bladder cancer model which is similar to human disease.^31^ The initiation of solid tumours originates from small numbers of dedicated stem cells called cancer stem cells. Keratin 14 (K14), which contains a nonhelical tail domain involved in K5–K14 large bundles self‐organising, has been shown to be overly expressed in basal‐like bladder cancer, and K14^+^ cell subpopulations serving as bladder cancer stem cells contribute to tumourigenesis and chemoresistance in vivo.^32^, ^33^, ^34^ In this study, we established conditional knockout mice to ablate Nat10 in K14^+^ bladder cancer stem cells to investigate the function and regulatory networks of the acetyltransferase NAT10 in vivo.

In our research, NAT10 was found to be highly expressed in BLCA and to participate in promoting tumour proliferation, metastasis and stemness maintenance by virtue of ac4C modification on binding target transcripts. We utilised a transcriptome‐wide acRIP‐sequencing (acRIP‐seq) approach to explore downstream genes directly bound by NAT10 and ac4C‐modified localisation in specific motifs. Then, we identified the ac4C enrichment of three targets directly bound to NAT10 that were notably reduced after knockdown of NAT10, resulting in a decrease in stability or translation within these genes. NAT10 is verified to contribute to proliferation and tumourigenesis in vivo via its effect of acetylation on downstream targets. Taken together, this study reveals the impact of NAT10‐mediated epi‐transcriptome on ac4C occupation within target genes which enhances stability and translation, potentially boosting the BLCA process.

## 2 RESULTS

### 2.1 Analysis of NAT10 expression and its prognostic value in BLCA

**FIGURE 1:**
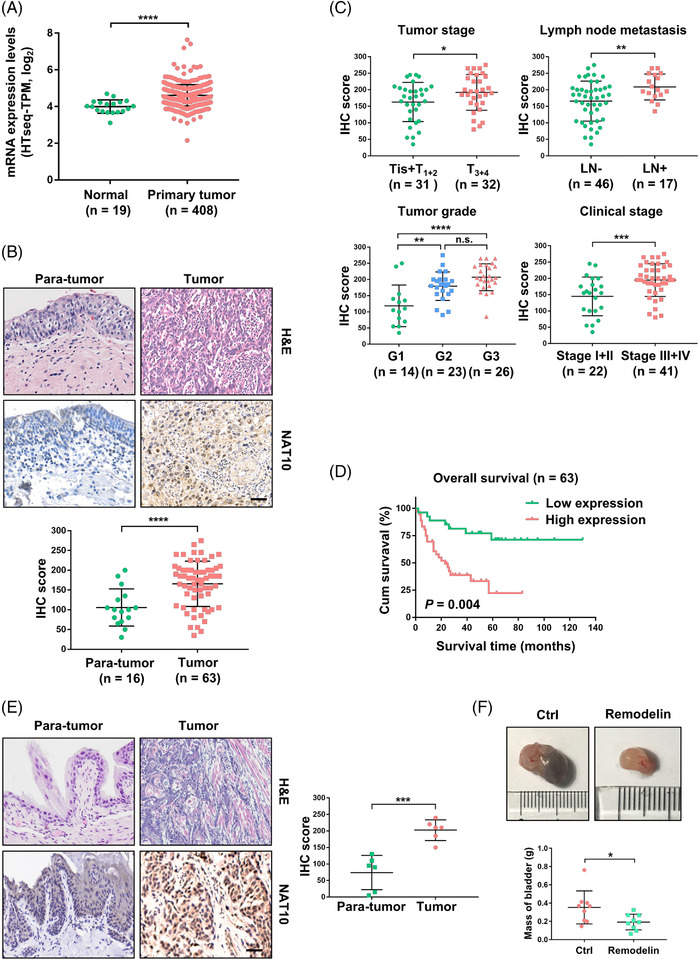
NAT10 is overexpressed in bladder urothelial carcinoma. (A) The mRNA level of NAT10 in bladder urothelial carcinoma based on TCGA dataset. (B) Representative H&E staining and IHC images of bladder cancer tissue microarrays to show the histology and expression of NAT10 in tumours or corresponding normal tissues. (C) The relationship between NAT10 expression and clinical features in patients with bladder cancer according to the tissue microarray. (D) Effect of NAT10 expression level on BLCA patient overall survival. (E) Representative H&E staining and NAT10 IHC images in mouse bladder cancer tissues and the corresponding normal tissues and the IHC score quantification (*n* = 6). Scale bars: 50 μm. (F) Representative images showing NAT10 inhibitor Remodelin significantly prevented bladder tumour growing (*p < .05, *n* = 9)

To characterise the expression of NAT10 in BLCA, we first investigated the published clinical dataset TCGA (The Cancer Genome Atlas) and found that NAT10 was expressed at a much higher level in BLCA tissues than normal tissues (Figure 1A). In addition, we examined the protein level of NAT10 using a commercial BLCA tissue microarray that included 16 pairs of tumour sections with adjacent normal tissues and 47 other tumour sections. Consistently, our immunohistochemistry (IHC) results from the tissue microarray samples demonstrated that the expression of NAT10 in tumours was higher than that in para‐tumour tissues (Figure 1B). Furthermore, patients with high levels of NAT10 carried more aggressive tumours and were more likely to develop lymph node metastases (Figure 1C). Patients with high levels of NAT10 had developed more heterotypic tumours and were more likely to progress into advanced stages (Stage III + IV) (Figure 1C). Kaplan–Meier curves with a log‐rank test showed that patients with low NAT10 expression had better overall survival, suggesting a possible role of NAT10 in predicting prognosis and promoting the progression of BLCA (Figure 1D). We found that NAT10 expression was not correlated with the age or sex of patients with BLCA, although a previous study showed the involvement of NAT10 in age‐related disorders (Figure S1A and B).^19^, ^24^ Consistent with the results from BLCA patients, NAT10 showed a higher level of expression in BBN‐induced mouse bladder cancer than in corresponding normal tissues (Figure 1E). To explore whether this elevated expression level of NAT10 has biological significance in the development of bladder cancer, we treated BBN‐induced BLCA mice with the NAT10‐specific inhibitor Remodelin for 4 weeks. Compared with the control group, the mice treated with Remodelin developed significantly smaller tumours (Figure 1F). Together, these data suggest that NAT10 might play a role in BLCA progression.

### 2.2 Inhibition of BLCA tumour progression through NAT10 disruption

**FIGURE 2:**
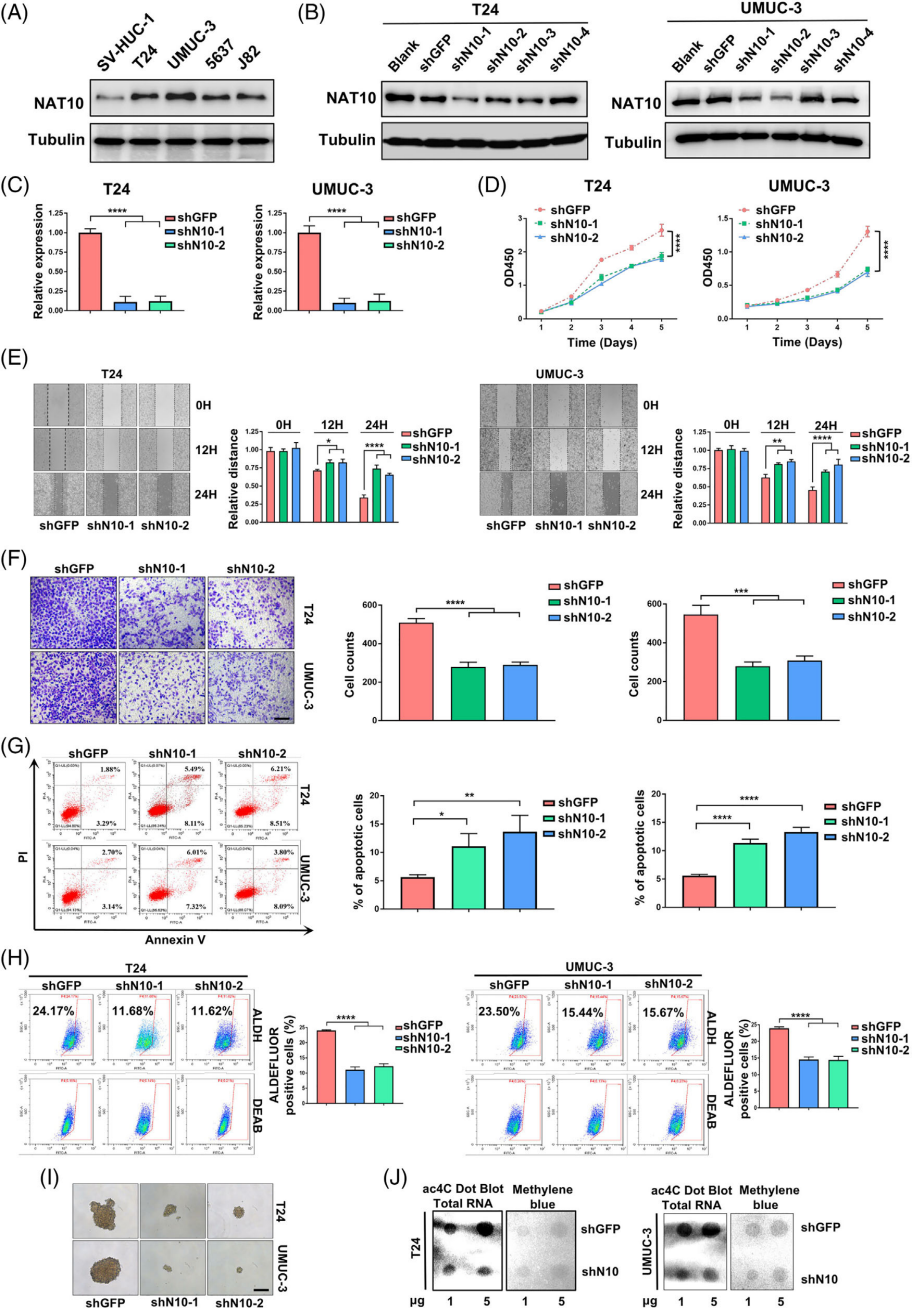
Knockdown of NAT10 suppresses tumourigenesis of BLCA cells. (A) Detection of NAT10 protein expression in uroepithelial cells and bladder cancer cell lines. (B) Validation of shRNAs against NAT10 knockdown efficiency by Western Blot in T24 and UMUC‐3 cell lines. (C) Determination of mRNA level after knocking down NAT10 by qPCR. The level of NAT10 mRNA is decreased by about 89% (shNAT10‐1) and 88% (shNAT10‐2) respectively in T24 cells. In UMUC‐3 cells, NAT10 mRNA is reduced by 90% (shNAT10‐1) and 88% (shNAT10‐2) (****p < .0001, *n* = 3). (D) Knockdown of NAT10 markedly inhibits proliferation of T24 and UMUC‐3 cells (****p < .0001, *n* = 3). (E) Migration capacity is significantly reduced follow by attenuation of NAT10 in bladder cancer cells (*p < .05, **p < .01, ****p < .0001, *n* = 3). (F) Downregulation of NAT10 weakens the ability of invasion in bladder cancer cell lines (****p < .0001, *n* = 3). (G) Inhibition of NAT10 induces the apoptosis of T24 and UMUC‐3 cells. The rate of apoptotic cells increases from 5.65% (shGFP) to 11.1% (shNAT10‐1) and 13.68% (shNAT10‐2) in T24 cells, while the proportion of apoptotic UMUC‐3 cells rises from 5.62% (shGFP) to 11.37% (shNAT10‐1) and 13.31% (shNAT10‐2) (*p < .05, **p < .01, ****p < .0001, *n* = 3). (H) Knockdown of NAT10 results in the reduction of cell stemness. With the use of shNAT10‐1 and shNAT10‐2 to block NAT10 expression, the percentage of ALDH^br^ T24 cells falls from 24.17% to 11.66% (shNAT10‐1) and 11.62 (shNAT10‐2). The rate of ALDH^br^ UMUC‐3 cells is decreased from 23.50% to 15.44% (shNAT10‐1) and 15.67% (shNAT10‐2) after knockdown of NAT10 (****p < .0001, *n* = 3; DEAB, diethylaminobenzaldehyde, an inhibitor for human ALDH). (I) Knockdown of NAT10 attenuated the ability of sphere formation in BLCA cells. Scale bars: 100 μm (*n* = 3). (J) Knockdown of NAT10 markedly inhibits total RNA ac4C abundance in T24 and UMUC‐3 cells

To explore the function of NAT10 in BLCA, we evaluated the protein level of NAT10 in SV‐HUC‐1 human uroepithelial cells and four different BLCA cell lines, T24, UMUC‐3, 5637 and J82. We found that the expression of NAT10 in J82 and 5637 cells was noticeably lower than that in T24 and UMUC‐3 cells (Figure 2A). We then used four different shRNAs against NAT10 to knockdown NAT10 expression in T24 and UMUC‐3 cells. Our data showed that shN10‐1 and shN10‐2 were able to inhibit the protein expression levels of NAT10 in both cell lines (Figure 2B). We also detected significantly lower NAT10 mRNA levels after shRNA knockdown (Figure 2C). The results from the CCK‐8 (cell counting kit‐8) assay showed that the proliferation ability of T24 and UMUC‐3 cells were significantly reduced by inhibiting NAT10 (Figure 2D). A wound‐healing assay revealed that NAT10 knockdown decelerated the healing of scratches in these cells (Figure 2E). Similarly, the invasion was reduced after inhibition of NAT10 in both T24 and UMUC‐3 cells (Figure 2F). Characterisation of shNAT10 cells revealed a higher ratio of apoptosis when compared with shGFP cells (Figure 2G). Based on previously reported methods for detecting stem cells, we next studied the requirement of NAT10 in maintaining the self‐renewal ability and found that the percentages of ALDH^br^ cancer stem cells decreased after disrupting their NAT10 expression (Figure 2H).^35^ In vitro the tumour‐sphere formation assays also demonstrated that knockdown of NAT10 attenuated sphere formation (Figure 2I). Moreover, dot‐blot assays showed reduced ac4C levels in total RNA after NAT10 deficiency (Figure 2J). These data suggest NAT10 that is required for BLCA tumourigenic properties.

### 2.3 Identification of NAT10‐mediated N4‐acetylation modification transcripts

**FIGURE 3:**
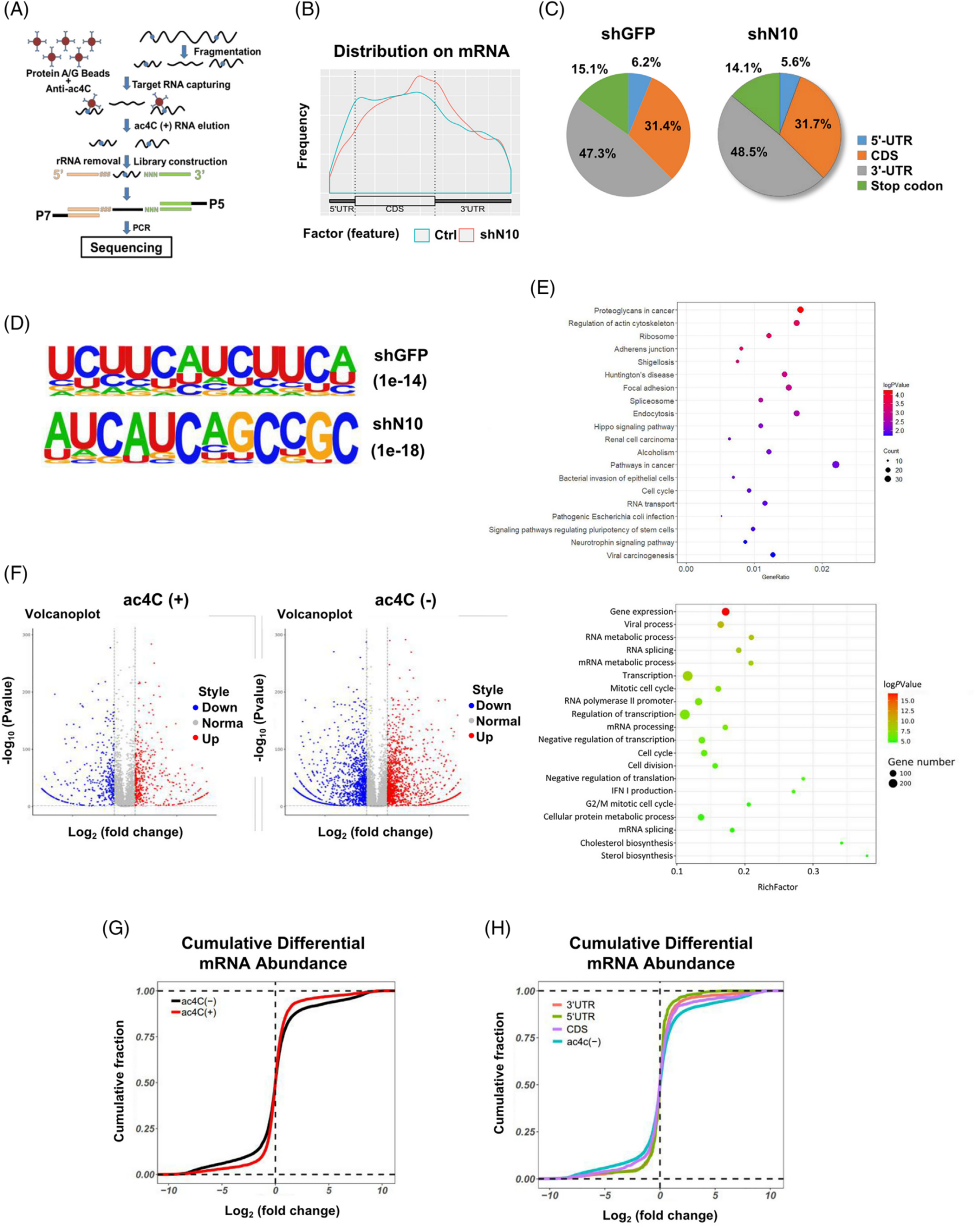
ac4C‐modified transcripts in BLCA cell lines are detected by acRIP‐sEquation (A) Schematic of acRIP‐seq for UMUC‐3 cells. (B) The distribution of ac4C‐containing peaks across mRNA in both control and NAT10 knockdown UMUC‐3 cells is displayed in metageneplot. (C) Representative pie chart of peak distribution exhibiting the proportion of total ac4C peaks in the indicated regions including 5′‐untranslated region (5′‐UTR), coding sequence (CDS), 3′‐ untranslated region (3′‐UTR) and stop codon. (D) Consensus motif of control and NAT10‐knockdown cells identified by HOMER. (E) Representative Pathway‐Analysis terms showing pathways of related genes significantly enriched by ac4C. (F) Volcano plots of differentially mRNA in control and NAT10 knockdown UMUC‐3 cells segregated by ac4C status. (G) Cumulative distribution function (CDF) plot showing differential expression of ac4C (–) or ac4C (+) transcripts in control and NAT10 knockdown UMUC‐3 cells. (H) CDF plot showing expression changes of protein‐coding genes in control and NAT10 knockdown UMUC‐3 cells for ac4C (–) and ac4C transcripts with peaks locate in the CDS, 5′UTR or 3′UTR

To investigate the potential ac4C‐modified mRNA targets regulated by NAT10 in BLCA, acRIP‐sequencing (acRIP‐seq) assay was conducted using mRNA isolated from control and NAT10‐knockdown UMUC‐3 cell lines (Figure 3A). In the control UMUC‐3 cells, over 4940 ac4C peaks were identified from acRIP‐seq libraries generated from UMUC‐3 cells, which agreed with 2167 transcripts. In the NAT10 knockdown UMUC‐3 cells, 1218 transcripts exhibited significantly decreased ac4C levels. The ac4C peaks in both the control and NAT10 knockdown cells were dispersed at the 3′‐UTR (47.3% vs. 48.5%), CDS (31.4% vs. 31.7%), stop codon (15.1% vs. 14.1%) and 5′UTR (6.2% vs. 5.6%) (Figure 3B and C). Overall, the decreased ac4C peaks in the NAT10 knockdown cells were mainly located near the stop codon region (Figure 3B and C). Consistent with previous research, the most common ac4C motif ‘CXXCXXCXX’ in both the control and NAT10 knockdown cells were significantly enriched in the ac4C peaks (Figure 3D). Gene Ontology analysis showed that genes with decreased ac4C peaks were related to several biological processes, such as cell–cell adhesion, mRNA decay, mRNA splicing, translation, stem cell maintenance and the cell cycle. Furthermore, KEGG Pathway‐Analysis results demonstrated that genes with decreased ac4C peaks were involved in pathways related to proteoglycans in cancer, adherens junctions, Shigellosis, the cell cycle and signalling pathways regulating the pluripotency of stem cells (Figure 3E).

According to a previous report, the acetylated status of transcripts is related to their abundance in HeLa cells.^10^ To examine whether this is the case in BLCA cells, we analysed the ac4C status and mRNA abundance. However, our results showed that transcripts with or without ac4C peaks both showed comparable up‐ or downregulated gene expression levels (Figure 3F). Additionally, ac4C‐modified transcripts, regardless of their ac4C peak location, showed no obvious sign of decreased transcript levels after the loss of NAT10 (Figure 3G and H). These data suggest that NAT10‐mediated mRNA ac4C modification might not be related to the overall mRNA abundance in BLCA cells.

### 2.4 mRNA ac4C modification enhances translation efficiency and increases stability of targets in BLCA cells

**FIGURE 4:**
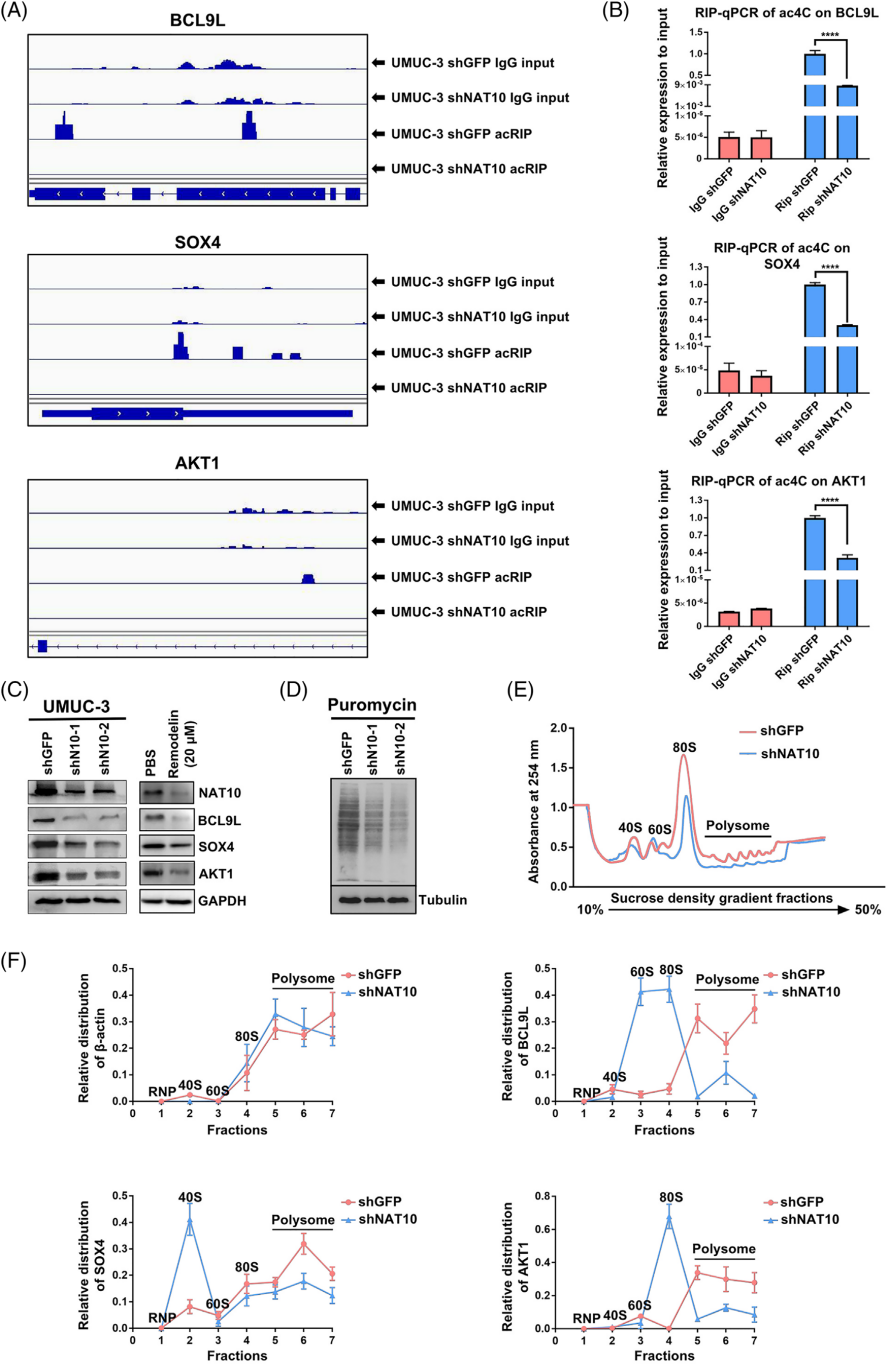
NAT10 regulates target genes in an N4‐acetylcytidine‐dependent manner. (A) Integrative Genomics Viewer (IGV) tracks displaying read distributions across target transcripts from acRIP‐sEquation. (B) Measuring the alteration of ac4C‐modified mRNA in BCL9L, SOX4 and AKT1 with or without knockdown of NAT10 through RIP‐qPCR (****p < .0001, *n* = 3). (C) Detection of BCL9L, SOX4 and AKT1 protein level by WB after blocking NAT10 expression. (D) Representative images of puromycin assay for determining the translation efficiency with Tubulin as equal loading control. (F) Polysome profile assay shows an overall decreased tendency of translation efficiency after knockdown of NAT10 in UMUC‐3 cells. (F) qPCR showing the changed relative distribution of BCL9L, SOX4 and AKT1 mRNA in different polysome gradient fractions between negative control and NAT10‐deficient cells. Beta‐actin without ac4C modification is used as control mRNA

Since the mRNA abundance is not necessarily globally affected by ac4C modification in BLCA cell lines, we reasoned it might affect the tumourigenic properties of BLCA cells via regulation of downstream target translation efficiency and/or stability. We then used Integrative Genomics View (IGV) software to search for potential downstream targets based on our acRIP‐seq datasets. We found that ac4C peaks in the genes associated with the cancer pathway (BCL9L), the cell cycle (AKT1) and stem cell maintenance (SOX4) decreased in the NAT10 knockdown UMUC‐3 cells (Figure 4A). To further validate our acRIP‐seq results, we performed acRIP‐qPCR assays and found that the ac4C abundances of BCL9L, SOX4 and AKT1 were notably reduced upon NAT10 knockdown in UMUC‐3 cells (Figure 4B). The mRNA half‐life assay demonstrated that the stability of BCL9L and SOX4 with highly abundant ac4C peaks was significantly reduced by downregulating NAT10, while AKT1 with a moderate abundance of ac4C displayed no significant change in mRNA stability with or without NAT10 deficiency (Figure S2A–C). Interestingly, Western blot results showed that BCL9L, SOX4 and AKT1 were markedly decreased at the protein level after NAT10 expression was blocked by shRNAs or Remodelin (Figure 4C). To examine whether NAT10 knockdown could lead to global inhibition of translation, we first performed a puromycin incorporation assay. Our results showed that puromycin incorporation efficiency was substantially inhibited in NAT10 knockdown whole cell lysates compared with the control cells (Figure 4D). In agreement with the puromycin incorporation assay, the results from the polysome fractionation assays demonstrated a reduction in translation efficiency in NAT10 knockdown cells (Figure 4E). Subsequently, we collected different sucrose gradients after centrifugation and performed qRT‐PCR to examine the distribution of these potential targets in different ribosome fractions. In the NAT10 knockdown cells, BCL9L, SOX4 and AKT1 mRNA were mainly found in monosomes or light polysomes, while these targets were detected mainly in the polysomes in the control cells (Figure 4F), suggesting a role of ac4C peaks in translational regulation.

### 2.5 BCL9L, SOX4 and AKT1 are functional targets of NAT10 in BLCA cells

**FIGURE 5:**
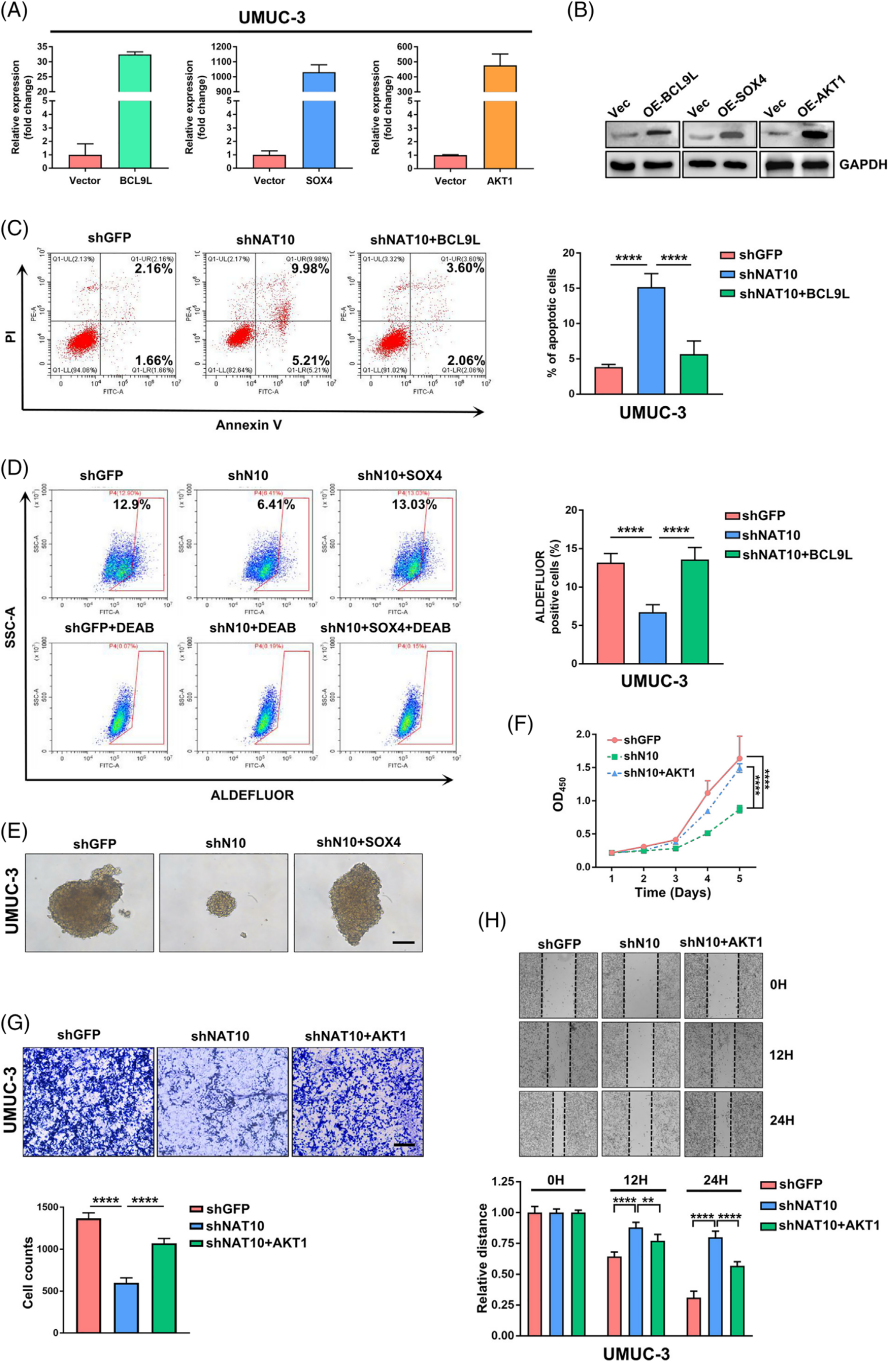
BCL9L, SOX4 and AKT1 partially restore cell phenotypes in NAT10‐depleted cells. (A) qPCR is used to measure the level of mRNA after transfection with BCL9L, SOX4 and AKT1 plasmids in UMUC‐3 cells. (B) Detection of BCL9L, SOX4 and AKT1 protein expression after transfection overexpressed plasmids by Western Blotting. (C) Apoptotic cell ratio in bladder cancer is tested using Annexin V‐FITC/PI kit. The stained cells are analysed by flow cytometry (****p < .0001, *n* = 3). (D) ALDEFLUOR assay is applied to assess the stemness of UMUC‐3 cells in control group, shNAT10 group, shNAT10 with overexpressed SOX4 group, respectively. Cells treatment with DEAB reagent is used as a negative control for ALDH^br^ staining. (****p < .0001, *n* = 3). (E) Transfection of SOX4 partially restores the sphere‐forming ability of UMUC‐3 cells after NAT10 knockdown. Scale bars: 100 μm. (F) The capacity of proliferation is detected using CCK‐8 assay under evaluation with OD450. (****p < .0001, *n* = 3). (G) Transwell experiments show that knockdown of NAT10 impairs invasion ability of UMUC‐3 cells while overexpression of AKT1 can partially restore cellular invasion capacity. (***p < .001, ****p < .0001, *n* = 3). (H) Transfection of AKT1 partially rescues the migration ability of UMUC‐3 cells after NAT10 knockdown through wound healing assay (**p < .01, ****p < .0001, *n* = 3)

To confirm the biological relevance of BCL9L, SOX4 and AKT1 in mediating NAT10 function in BLCA, we performed rescue experiments using pCDNA 3.1 (+)‐BCL9L, pCDNA 3.1 (+)‐SOX4 or pCDNA 3.1 (+)‐AKT1 recombinant plasmids (Figure 5A and B). Our results showed that the higher apoptotic rate of shNAT10 UMUC‐3 cells was restored after delivering recombinant plasmids containing BCL9L to the UMUC‐3 cells (Figure 5C). To validate whether the re‐expression of SOX4 could rescue stemness after disrupting NAT10, we conducted an ALDEFLU assay to identify ALDH^br^ cells and found that re‐expression of SOX4 restored the stemness of UMUC‐3 cells (Figure 5D). Similarly, re‐expressed SOX4 rescued the effects of reduced tumour‐sphere formation capacity after the suppression of NAT10 (Figure 5E). Furthermore, we found that the proliferation ability of UMUC‐3 cells was reduced followed by the disruption of NAT10 but was partially rescued by re‐expressing AKT1 (Figure 5F). Moreover, overexpression of AKT1 partly recovered the cell's invasive ability after the inhibition of NAT10 (Figure 5G). Likewise, the migration capacity was restored by overexpressing AKT1 (Figure 5H). Together, these findings demonstrate that NAT10‐dependent ac4C modification of target mRNAs functionally regulates the biological processes of BLCA cells.

To evaluate the clinical value of targets governed by NAT10‐mediated ac4C modification, we detected the protein expression level of downstream targets in tissue microarrays. Then, we analysed the correlation between the expression patterns of these target genes and clinical features, including tumour stage, lymph node metastasis status, tumour grade, clinical stage and overall survival. As shown in Figure S3A and B, BCL9L was abnormally highly expressed in BLCA patients. These patients were separated into two groups depending on their tumour stage, lymph node metastasis status and clinical stage, and three groups according to their tumour grade. Based on the analysis of our data, BCL9L expression was positively correlated with tumour invasiveness (tumour stage), metastatic ability (lymph node metastasis), cellular pleomorphism (tumour grade) and tumour progression (clinical stage) (Figure S3C). Survival curves showed that patients with high levels of BCL9L had a significantly shorter survival period than those with a low expression of BCL9L, suggesting that BCL9L might serve as a promising indicator for predicting the prognosis of patients with BLCA (Figure S3D). Subsequently, we found that the tumours expressed SOX4 at much higher levels when compared with the adjacent normal tissue (Figure S4A and B). Tumours in patients with higher expressions of SOX4 tended to be more invasive and were more likely to develop lymph node metastasis (Figure S4C). Furthermore, patients with grade 3 bladder cancer frequently expressed SOX4 at higher levels than those with lower‐grade tumours (Figure S4C). The SOX4 level did not, however, vary between grade 1 and grade 2 tumours. The overall level of SOX4 in patients with advanced cancer was much higher than that in patients in early stages (Figure S4C). A high expression of SOX4 was significantly associated with poor prognosis based on survival analysis (Figure S4D). Next, we explored the prognostic significance of AKT1, whose mRNA was modified with moderate enrichment of ac4C peaks. The IHC score of AKT1 remarkably increased at the protein level in bladder tumours compared with adjacent tissue (Figure S5A and B). However, the expression level of AKT1 was not related to the clinical parameters in patients with BLCA (Figure S5C). There was also no significant difference in overall survival between patients with low and high AKT1 expression (Figure S5D).

To verify our findings that BCL9L, SOX4 and AKT1 were direct targets modulated by NAT10, a co‐expression analysis in patient samples was carried out to verify a correlation between NAT10 and these three target genes. Consistent with our experiments for validating downstream genes in vitro, the expression of NAT10 was positively related to the expression of BCL9L (*r* = 0.5514, *p* < .0001), SOX4 (*r* = 0.6826, *p* < .0001) and AKT1 (*r* = 0.2702, *p* = .0322) (Figure S6A–C).

### 2.6 Promotion of BLCA growth via exogenous NAT10 expression

**FIGURE 6:**
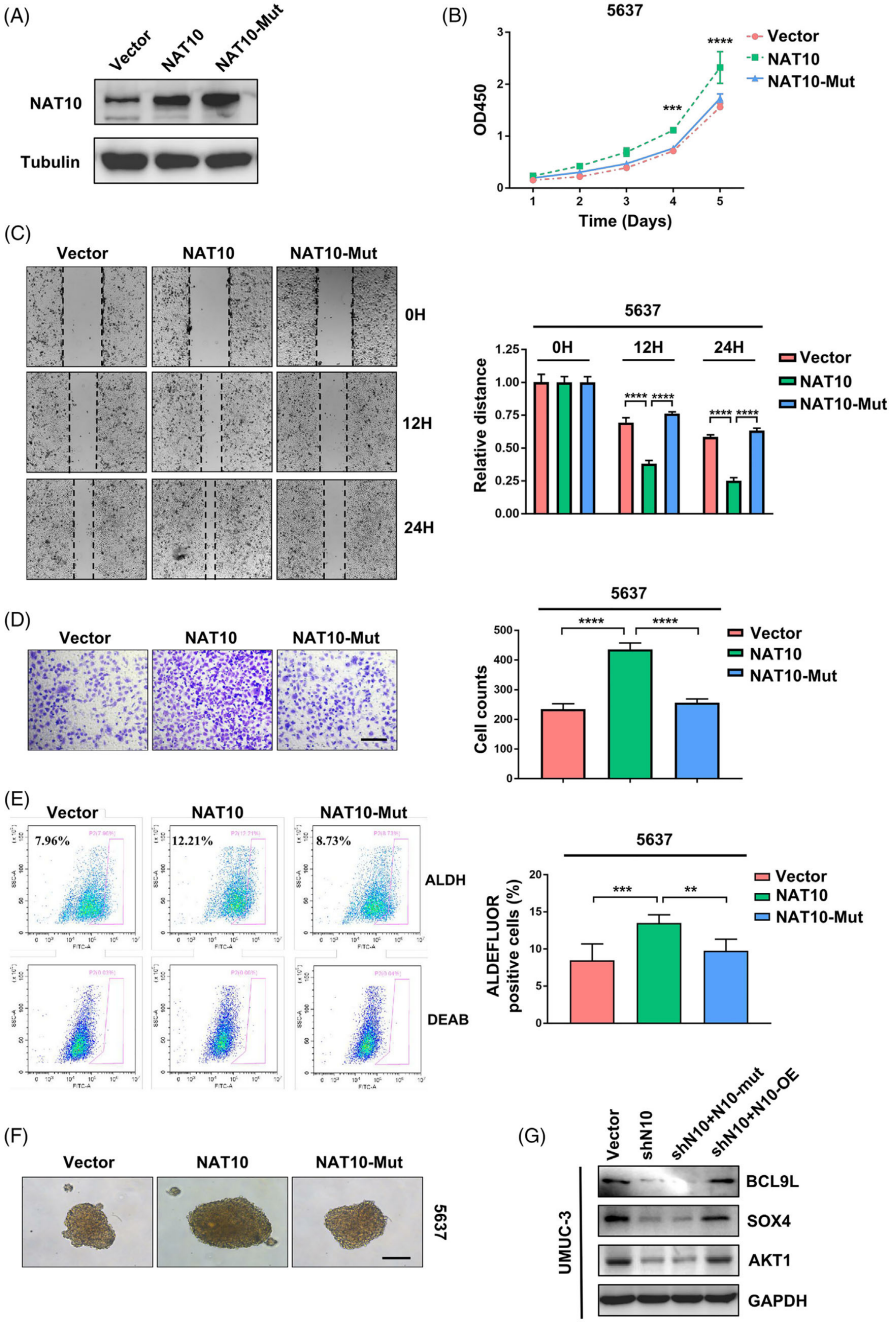
Overexpression of NAT10 promotes tumourigenic ability of 5637 cells. (A) The expression of NAT10 is examined after transfection of plasmids containing NAT10 sequence by Western Blot. (B) CCK‐8 kit is utilised to determine the proliferation in 5637 cells transfected vector, NAT10‐overexpressed plasmid and NAT10‐mutant‐overexpressed plasmid respectively (***p < .001, ****p < .0001, *n* = 3). (C) Wound healing assay demonstrate that forced expression of NAT10 but not NAT10‐mutant promotes the ability of migration of 5637 cell line (****p < .0001, *n* = 3). (D) In vitro transwell assay showing NAT10 increases invasiveness of 5637 cells (****p < .0001). (E) NAT10 contributes to the improvement of stemness in 5637 cells is determined by in vitro ALDEFLUOR assay (**p < .01, ***p < .001, *n* = 3). (F) NAT10 overexpression promotes the sphere‐forming ability of 5637 cells. Scale bars: 100 μm. (G) Western blot is used to test the level of downstream targets after overexpression of NAT10 or NAT10‐mutant in shN10 UMUC‐3 cells

To further validate the biological function of NAT10 in bladder cancer cells, we exogenously expressed NAT10 in 5637 cells, which had low NAT10 expression, using pICE‐FLAG‐NAT10‐siR‐WT (NAT10) or pICE‐FLAG‐NAT10‐siR‐G641E (NAT10‐mutant, mutated conserved glycine residue 641 to glutamate, which impaired the acetyl‐CoA binding structure of NAT10).^19^ Our results showed that the proliferation ability of the 5637 cells was dramatically increased by overexpressing of NAT10 but not the overexpression of the NAT10 mutant (Figure 6A and B). The wound‐healing assay indicated that the overexpression of NAT10 instead of the NAT10 mutant accelerated the healing of scratches and enhanced cell invasion in the 5637 cells (Figure 6C and D). In addition, the proportion of ALDH^br^ cells significantly increased after overexpressing NAT10 in the 5637 cells while overexpression of NAT10 could not restore stem‐cell‐like properties (Figure 6E). A similar phenomenon was observed for 5637 cells in which NAT10 was over‐expressed but not the NAT10‐mutant, in which the cells had increased sphere formation ability (Figure 6F). To thoroughly understand the regulatory role of NAT10‐mediated ac4C in modulating target mRNAs, plasmids encoding NAT10 or NAT10 with silenced acetylation binding sites were delivered to UMUC‐3 cells. The levels of BCL9L, SOX4 and AKT1 were returned by re‐expressing NAT10 but not the NAT10‐mutant after inhibition of NAT10 (Figure 6G). Collectively, our data demonstrated the essential role of NAT10 in promoting BLCA progression and CSC maintenance.

### 2.7 Repression of proliferation and tumourigenesis through NAT10 inhibition in vivo

**FIGURE 7:**
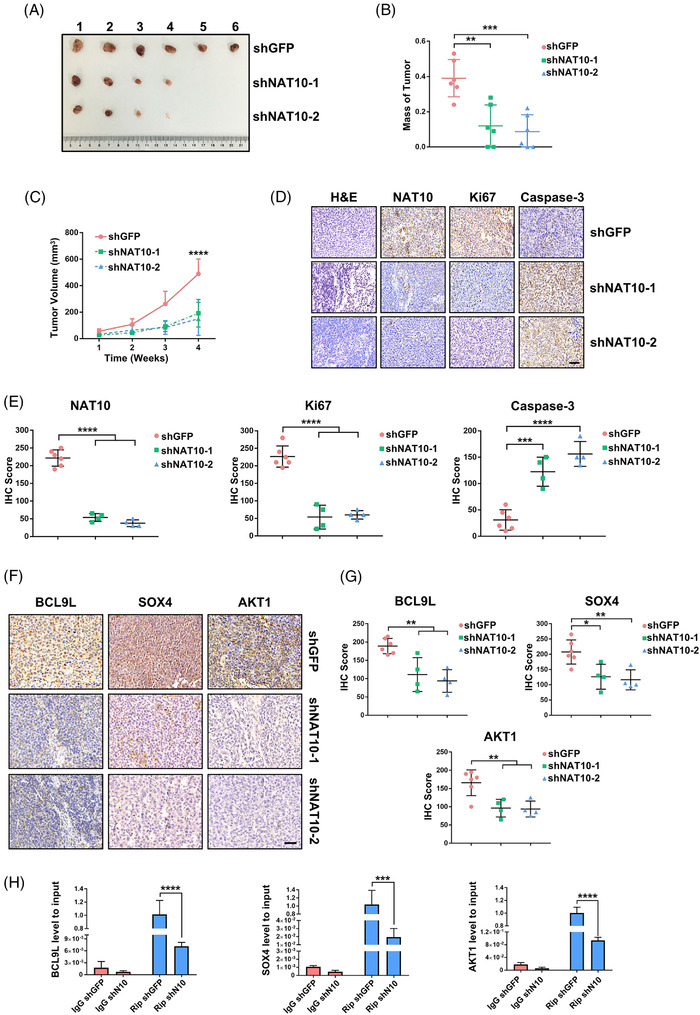
Establishment of the xenograft mouse model validates that NAT10 facilitates growth and progression of BLCA. (A) Tumour images of xenograft nude mice model subcutaneously injected with UMUC‐3 cells. (B) Mass of tumours in shGFP, shNAT10‐1 or shNAT10‐2 group at the endpoint is shown (**p < .01, ***p < .001, *n* = 6). (C) Tumour volume is recorded from the first week, once a week for a total of 4 weeks (**p < .01, ****p < .0001, *n* = 6). (D) Representative images for H&E and IHC staining in nude mouse sections. Scale bars: 50 μm. (E) IHC score is computed according to immunostaining intensity and frequency on xenograft model sections (***p < .001, ****p < .0001, *n* = 6). (F) IHC staining showing the expression level of targets regulated by NAT10 in vivo. Scale bars: 50 μm. (G) The level of BCL9L, SOX4 and AKT1 is evaluated based on IHC staining (**p < .01, *n* = 6). (H) The level of BCL9L, SOX4 and AKT1 mRNA enriched by ac4C is examined by Rip‐qPCR experiments (***p < .001, ****p < .0001, *n* = 6)

To investigate the role of NAT10 in regulating proliferation and tumourigenesis in vivo, we injected 1 000 000 viable UMUC‐3 cells transduced with shGFP, or shNAT10 into BALB/c nude mice. The mice injected with shGFP‐UMUC‐3 cells displayed a significantly stronger proliferation and tumourigenicity than to those injected with shNAT10‐UMUC‐3 cells (Figure 7A). Notably, compared with shGFP‐UMUC‐3 tumour‐bearing mice, shNAT10‐1 and shNAT10‐2‐UMUC‐3 tumour‐bearing mice demonstrated a reduced tumour burden and tumour growth (Figure 7B and C). Additionally, we found a significant decrease in NAT10 and Ki67 (proliferation marker protein) levels and a significant increase in Caspase‐3 (apoptosis execution‐related protein) levels in tumour cells in vivo (Figure 7D and E). Next, we explored whether the depletion of NAT10 could lead to the changed expression pattern of targets modified by ac4C in vivo. In accord with the findings in vitro, the staining frequency and intensity of BCL9L, SOX4 and AKT1 decreased significantly in downregulated‐NAT10 xenograft tumours (Figure 7F and G). To further explore whether these targets were regulated by ac4C modification, xenograft tumour samples were used for RNA extraction and Rip‐qPCR. Likewise, the Rip‐qPCR experiment showed that ac4C‐enriched BCL9L, SOX4 and AKT1 declined significantly following the inhibition of NAT10 (Figure 7H). In general, in agreement with the phenotypes found in vitro, inhibition of NAT10 attenuated tumourigenicity via suppression of the target genes BCL9L, SOX4 and AKT1 by means of ac4C peak impairment in the NAT10‐depleted mouse model.

### 2.8 Ablation of Nat10 in K14^+^ cells inhibits BLCA development

**FIGURE 8:**
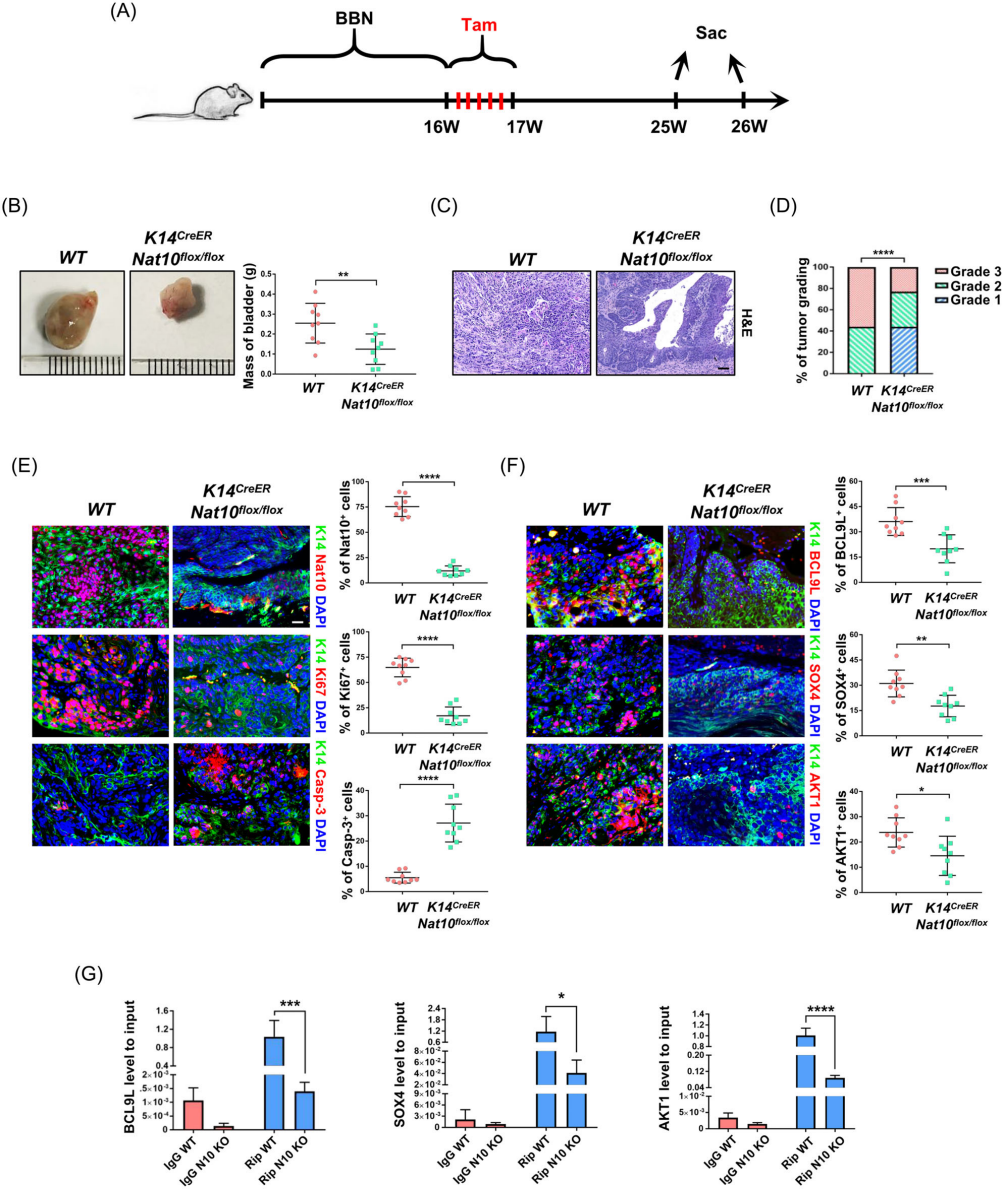
Ablation of Nat10 in K14+ cancer stem cells restrains BLCA progression. (A) Schematic diagram showing the experimental procedures for induction of BLCA and conditional knockout of Nat10 in transgenic mice. (B) Representative images for bladder tissues from WT or Nat10 KO mice showing the effect of Nat10 deletion on the tumour burden (**p < .01, *n* = 9). (C) H&E staining of wild‐type and *K14^CreER^*; *Nat10^flox/flox^* mice showing the bladder cancer histological patterns. Scale bars: 100 μm. (D) Tumour grading statistics indicates that knockout of Nat10 in K14 expressing cells delays bladder cancer progression (****p < .0001, *n* = 9). (E) Immunofluorescent staining of K14 (green), NAT10, Ki67, Caspase‐3 (red) and DAPI (blue) in WT and Nat10 knockout mice respectively. Scale bars: 50 μm (****p < .0001, *n* = 9). (F) Representative images for immunostaining of K14 (green), BCL9L, SOX4, AKT1 (red) and DAPI (blue) in wild‐type and *K14^CreER^; Nat10^flox/flox^* mice respectively (*p < .05, **p < .01 and ***p < .001, *n* = 9). (G) RIP‐qPCR experiments were applied to detect the changes of ac4C‐modified targets with or without deficiency of Nat10 in mice carrying bladder cancer (*p < .05, ***p < .001 and ****p < .0001, *n* = 9)

To reinforce our findings, we crossed *K14^CreER^* mice with *Nat10^flox/flox^* mice to generate *K14^CreER^; Nat10^flox/flox^* (NAT10‐cKO) mice, which allowed us to conditionally delete Nat10 in K14^+^ cancer stem cells. Bladder cancer in wild‐type and NAT10‐cKO mice was induced by continuously supplementing their drinking water with BBN for 16 weeks. Then, intraperitoneal injection of tamoxifen was applied for inducing the knockout of Nat10, and the mice were treated with normal water for another 10 weeks before sample collection (Figure 8A). Compared with the wild‐type mice, NAT10‐cKO mice carried a lower tumour burden (Figure 8B). Measurements of the bladder tissues with tumours demonstrated a significant reduction in bladder mass after the deletion of Nat10 in K14^+^ cells (Figure 8B). H&E staining of tumour sections showed that wild‐type mice developed a higher grade of malignant tumours when compared to the NAT10‐cKO mice (Figure 8C). Tumour grading statistics in both the control and the NAT10‐cKO groups indicated that the loss of Nat10 in bladder cancer stem cells effectively delayed cancer progression (Figure 8D). In addition, deficiency of Nat10 in K14^+^ cells resulted in decreased levels of Ki67 and elevated Caspase‐3, indicating that Nat10 was responsible for proliferation promotion and apoptotic inhibition in vivo (Figure 8E). Consistent with the results in vitro and from the nude mouse xenograft model, immunofluorescence staining of specimens collected from our genetically engineered mice showed that the depletion of Nat10 in bladder cells expressing K14 also led to the downregulation of BCL9L, SOX4 and AKT1 (Figure 8F). We hypothesise this is caused by NAT10‐mediated ac4C modification. To verify our hypothesis, bladder tissues obtained from wild‐type and NAT10‐cKO mice were ground and homogenised for RNA extraction. RIP‐qPCR was conducted using an anti‐ac4C antibody to detect the abundance of ac4C‐modified mRNA fragments and found that ac4C peaks upon BCL9L, SOX4 and AKT1 significantly decreased as a result of the Nat10 knockout (Figure 8G). In this section, we utilised an animal model that is more analogous to human bladder cancer to ultimately substantiate the role of NAT10 in regulating the expression of targets such as BCL9L, SOX4 and AKT1 through ac4C modification, therefore, promoting BLCA progression.

## 3 DISCUSSION

Epitranscriptome has emerged as an important field for early‐stage diagnostics and the treatment of cancer. In this study, we found that if NAT10 is highly expressed in BLCA, this can be an indicator for predicting a poor prognosis accompanied by lymph node or distant metastasis. In addition, a functional study confirmed that NAT10 is required for cell proliferation, invasion and stem cell‐like properties, uncovering the oncogenic role of NAT10 in BLCA. Both the NAT10‐specific antagonist and a genetic knockdown of NAT10 can impede BLCA progression, which indicates that targeting NAT10 might be a novel strategy for treating BLCA.

mRNA is a substrate for a series of epigenetic modification‐related compounds delivered to ribosome‐protein complexes. This study demonstrates that the overexpression of NAT10 instead of the NAT10‐mutant, whose active enzyme site is silenced, could enhance the malignancy of 5637 cells, reinforcing the hypothesis that the acetylation activity of NAT10 is correlated with the degree of malignancy of BLCA. Moreover, exogenous NAT10, but not the mutant NAT10, recovered the decline in BCL9L, SOX4 and AKT1 caused by the repression of NAT10. These results reinforce the theory that NAT10‐dependent ac4C modification is crucial for bladder cancer progression, supplying a comprehensive understanding of the elaborate regulatory mechanism of the epitranscriptome in BLCA. The NAT10‐dependent epitranscriptome may be a dynamic equilibrium process based on the intracellular CoA/acetyl‐CoA ratio.^36^ Previous studies have proven that NAT10‐mediated ac4C modification is dynamic and reversible and involves various aspects of biological processes, such as rRNA biogenesis and translation.^10^, ^37^, ^38^, ^39^, ^40^ The precise ways in which NAT10‐mediated ac4C promotes a target gene's stability and translation efficiency remains unclear, despite a report that proposes an optimised platform for profiling interacting proteins in acetylation.^40^ In recently published studies, NAT10 has been verified to interact with THUMP domain‐containing protein 1 (THUMPD1), acting as a component‐adaptor compound to modify tRNA acetylation in eukaryotes.^22^ In addition, another study indicated that NAT10‐mediated transcriptional repression of Che‐1 can be interrupted by means of deacetylation of NAT10 by Sirt1.^41^ Our data suggest the characterisation of NAT10 as an enzyme in ac4C modification by directly binding to the sequence‐characterised regions of BCL9L, SOX4 and AKT1 to support bladder tumourigenesis. NAT10‐mediated ac4C modification is bound up with protein processing, gene expression and protein binding. BCL9L and SOX4, but not AKT1, are of prognostic value in predicting outcomes for patients with BLCA, providing further evidence for the role of NAT10 in estimating the progression of the disease. However, mRNAs with ac4C or without ac4C peaks display no significant differences in their overall abundance, which is in contrast to a previous study. Our study hypothesises that the biological function of ac4C modification is likely to be context‐dependent.

In BLCA, the NAT10 knockout results in the instability of target genes, which is probably caused by a decreased ac4C peak. More importantly, BCL9L, SOX4 and AKT1 were substantially reduced at the protein level, indicating that post‐transcriptional ac4C modification mediated by NAT10 can influence gene expression in many respects. Indeed, the requirement of BCL9L for activating Wnt signalling indicates an essential role in colon cancer and hepatocyte transformation.^26^ Nevertheless, the different functions of SOX4 in TGF‐β‐driven tumourigenesis or apoptosis in pancreatic ductal adenocarcinoma demonstrate its multiple and various effects based on context.^28^ The transforming E17K PH domain mutation that occurs in AKT1 is confirmed to be associated with breast, colorectal and ovarian cancers.^42^ To summarise, the dynamic accommodation of BCL9L, SOX4 and AKT1 transcripts mediated by NAT10‐dependent ac4C modification is critical for the progression of bladder cancer.

Bladder cancer is diagnosed in approximately 550 000 people, of which approximately 200 000 die from it worldwide each year, making it the 12th most common malignancy.^43^ Hence, further study to define biological indicators and effective treatment strategies is urgently needed. In conclusion, we demonstrate that the loss of NAT10 downregulates mRNA expression by weakening stability and translation, potentially repressing BLCA progression. Targeting the catalyst‐dependent epitranscriptome by NAT10‐mediated ac4C occupation within target genes shows great benefits in cancer molecular therapy.

## 4 MATERIALS AND METHODS

### 4.1 Mice

Six‐ to eight‐week‐old nude mice for the xenograft model were obtained from GemPharmatech (Jiangsu, China) and housed in specific pathogen‐free conditions. Approximately 1 × 10^6^ bladder cancer cells were injected into the backs of mice subcutaneously. The tumour sizes in the mice were measured weekly, and the mice were kept for 4 weeks before being euthanised and sampled. For induction of BLCA, 6‐ to 8‐week‐old transgenic mice were fed with water adding 500 μg/ml BBN for 16 weeks and then fed with normal water for another 9–10 weeks. *K14^CreER^* transgenic mice (JAX stock: 005107) and *C57BL/6J* genetic background mice were purchased from the Jackson Laboratory. *Nat10^flox^* genetically engineered mice were generated using the CRISPR‐Cas9 genetic knockout system. The BBN‐induced bladder cancer mice were given Remodelin by oral gavage at 0.1 mg/g per day for 4 weeks. For inducing the knockout of Nat10 in K14^+^ cells, Cre‐recombinase was activated by intraperitoneal injection of tamoxifen at a dose of 0.225 mg per 1 g body weight once a day for 5 days. All work of the animal was approved by the Institutional Animal Care and Use Committee, Zhujiang hospital, Southern Medical University.

### 4.2 Cell lines

Human uroepithelial SV‐HUC‐1 cell and bladder cancer cell lines (T24, UMUC‐3, J82 and 5637) were purchased from the Type Culture Collection of the Chinese Academy of Sciences in Shanghai, China. The SV‐HUC‐1 cell was cultured in Ham's serum‐supplemented F‐12K (Gibco, USA) medium. The T24 and the 5637 cells were cultured in RPMI‐1640 (Gibco, USA) medium supplemented with 10% fetal bovine serum (Gibco, USA), while the UMUC‐3 cell line was cultured in basic DMEM (Gibco, USA) supplemented with 10% FBS, and the J82 cells were cultured in NEAA MEM supplemented with 10% FBS. All of the cell cultures were maintained at 37°C with 5% CO2. The shRNAs in the pLKO.1 lentiviral vector designed by The RNAi Consortium (TRC) were obtained from horizon™ and used for targeting NAT10. Stable transfections of shRNAs were performed with Lipofectamine™ 2000 transfection reagent according to the manufacturer's instructions. The sequences of shRNAs were as follows: shNAT10‐1, 5′‐ AACAGGAACAAATCCAGCTCG ‐3′; shNAT10‐2, 5′‐ ATAGTCACTGTGTACAATTGC ‐3′; shNAT10‐3, 5′‐ AATTCAGGATTTAGAGACTGG ‐3′ and shNAT10‐4, 5′‐ TACGGTGTGAATTTCCTGTGG ‐3′.

### 4.3 Primary bladder cancer samples

The bladder tissue microarray was commercially purchased from SHANGHAI OUTDO BIOTECH CO., LTD. and contained samples donated from biopsies and surgeries to be used in this current study by patients between 2007 and 2012. For the NAT10 expression studies, 63 bladder cancer samples and 16 corresponding non‐tumour normal tissues were collected from patients whose ages ranged from 42 to 85 years.

### 4.4 Plasmid construction

pICE‐FLAG‐NAT10‐siR‐WT and pICE‐FLAG‐NAT10‐siR‐G641E plasmids for the overexpression of NAT10 or NAT10‐mut were obtained from Addgene (#59365 and #59366). Plasmids encoding BCL9L, SOX4 and AKT1 mRNA were commercially purchased from IGE Biotechnology LTD. (Guangzhou, China) according to the GenBank NCBI reference sequences NM_001378213.1 and NM_003107.3.

### 4.5 acRIP‐seq

For acRIP‐seq analysis, 150 μg of total RNA from parental and shNAT10 cells was purified with an RNA extraction kit. Purified samples were fragmented into appropriately 200 nt pieces by a 10× fragmentation buffer (100 mM Tris‐HCl, 100 mM ZnCl2 in nuclease‐free H2O) and incubated at 70°C for 6 min. Then, EDTA was immediately added to stop the reaction. A Zymo RNA clean and concentrator‐25 kit was applied to further purify and recover fragmented RNA. Anti‐N4‐acetylcytidine (ac4C) and control rabbit IgG antibodies were used to interact with the ac4C modification sites on RNA. The antibody‐conjugated samples were incubated with prewashed Protein G Dynabeads at 4°C for 4–6 h, and immunoprecipitation products were recovered with a HiPure cell miRNA Kit. A QIAseq FastSelect –rRNA HMR Kit (Cat. No. 334376) was applied for rapid rRNA removal. The isolated and purified products were used for the input libraries constructed (parental versus shNAT10 of IgG) and acRIPs (parental versus shNAT10 of anti‐ac4C) with an Epi™ mini longRNA‐seq kit (Epibiotek, cat. No. R1815) according to the manufacturer's instructions. A Bioptic Qsep100 Analyzer was used for library quality control. Libraries were multiplexed on a NovaSeq high‐throughput sequencing platform using PE150‐sequencing mode.

### 4.6 Identification of ac4C sites and distribution in UMUC‐3 cells

Samples from the total RNA of the UMUC‐3 cells were preprocessed to remove low‐quality bases and adaptor sequences with the use of FastQC. We conducted unique mapped reads to check the conformity of the raw data. To this end, HISAT2 software was used to compare the filtered effective sequencing data with the reference genome to obtain the file in BAM format, and then the comparison rate was calculated. Data with a ratio of more than 60% were considered eligible. Next, reads obtained by acRIP‐seq were aligned to the genome to find the enrichment of these short sequences in the genome with the ExomePeak package in R, and the metageneplot was drawn based on the identified ac4C peaks.

To analyse the location of ac4C peaks within transcripts or transcript features (CDS and UTR), the relative CDS and UTR were resolved from annotation BED files and intersected with summit positions from the Hypertext editor. Reads from raw data were managed and aligned to the GRCh38/hg38 described for acRIP‐seq. Differential expression of the ac4C‐targeted mRNAs was calculated using DESeq2. Downstream analysis of the targets was processed within genes with protein‐coding annotation according to the Ensembl gene ID annotation. According to the acRIP‐seq results, genes were divided into two categories, acetylated (ac4C+) or non‐acetylated (ac4C‐), and merged with DEseq2 output for log_2_‐fold expression change in the negative control compared to the NAT10‐knockout UMUC‐3 cells. Genes with the ac4C modification were further divided into three categories based on the position of their ac4C peaks, and the log_2_ fold expression differences were computed and plotted for the cumulative distributions of mRNA abundance using ggplots package and Guitar package in R. The significance of difference was verified by the Kolmogorov‐Smirnov (KS) test.

### 4.7 Gene Ontology and KEGG Analysis

Gene Ontology classification and KEGG pathway analysis were performed using ToppGene Suite (https://toppgene.cchmc.org/enrichment.jsp) and the KEGG PATHWAY Database (https://www.kegg.jp/kegg/pathway.html). An adjusted *p* value less than .05 was taken as indicating statistical significance for Gene Ontology and KEGG Analysis.

### 4.8 Immunohistochemistry

The bladder cancer and the corresponding non‐tumour tissues were cut into 4–6 μm sections. The slides were deparaffinised with dimethylbenzene for 20 min; the samples were hydrated in different gradients of ethanol. Endogenous peroxidases were blocked by incubation for 10 min in 3% H_2_O_2_. After blocking with 5% skim milk, the antigen retrieval for NAT10 was performed with the use of ethylenediaminetetraacetic acid (EDTA) buffer (Beyotime Biotechnology, Shanghai, China) for 21 min. After the sections were wiped dry, the NAT10 antibody (1:200; Abcam, Cambridge, UK) was applied at 4°C for 8–12 h. Then, the slides were incubated with an anti‐rabbit secondary antibody for half an hour at room temperature in a wet box. After incubation, dripped DAB (ZSGB‐BIO, Beijing, China) was applied for 3–5 min. The sections were lightly counterstained with hematoxylin. The percentage of positive staining (frequency) was scored as follows: 0 for no cells with positive staining; 1–100 for 1%–100% of cells stained. The staining intensity (intensity) of the cells was scored as 0 for not stained, 1 for weakly posotive, 2 for moderately stained and 3 for strongly positive. The IHC scores (0–300) were the product of the frequency and intensity scores. Both the frequency and intensity were assessed in a double‐blind manner.

### 4.9 Immunofluorescence

Prepared slides were dewaxed in the hood for 10 min and then soaked in 4% paraformaldehyde for another 5 min. The slides were washed with PBS three times for 5 min each. Then, the samples were transferred to a plastic jar with a citrate buffer and microwaved for 7–8 min with an on‐place lid in a 1‐L beaker filled with water to the 500 ml mark. Slices were cooled at room temperature in a citrate buffer for 30 min and then the citrate was dumped before adding 1× PBS to wash the sections for 5 min twice. Sections were blocked in a blocking buffer (PBS plus 1% normal donkey serum, 1% BSA and 0.2% Triton‐X) at room temperature within humidified chambers for 30 min. Then, the blocking buffer was discarded, and the diluted primary antibody (1:100) was added to each side. All tissue sections were incubated at 4°C for 8–12 h. The next day, a Highly Cross‐Adsorbed Secondary Antibody (Alexa Fluor Plus 488 or 594, ThermoFisher) was added to wash the slides and incubated for 1 h at room temperature in a humidified box. The sections were washed twice with PBS for 5 min, and DAPI (5 μg/ml) was applied to stain the nuclei for 2 min. Images were taken with a forward fluorescence microscope (Nikon).

### 4.10 RNA isolation and qPCR

To investigate the function of NAT10, total RNA was isolated with the TRIzol reagent (Invitrogen Life Technologies, USA), and the reverse‐transcribed reaction was performed with a reverse‐transcription kit from Takara Biotechnology Co., Ltd. (Dalian, China). The reverse transcription‐PCR conditions were set according to the manual instruction. The designed primers for the qPCR were as follows: NAT10 (forward), 5′‐ ATAGCAGCCACAAACATTCGC ‐3′; NAT10 (reverse), 5′‐ ACACACATGCCGAAGGTATTG ‐3′; GAPDH (forward), 5′‐ TGTGGGCATCAATGGATTTGG ‐3′ and GAPDH (reverse), 5′‐ ACACCATGTATTCCGGGTCAAT ‐3′. The designed primers for the acRIP‐qPCR were as follows: BCL9L (forward), 5′‐ GTCATGAATCGCTCCGCCTC ‐3′; BCL9L (reverse), 5′‐ GACCCAGCAATTTTGCCCAG ‐3′; SOX4 (forward), 5′‐ CTCGACCGGGACCTGGATTT ‐3′, SOX4 (reverse), 5′‐ AGATCATCTCGCTCACCTCG ‐3′; AKT1 (forward), 5′‐ GTGAATATGCGGGGAGCAGC ‐3′ and AKT1 (reverse), 5′‐ GAGGACGCCAAGGAGATCAT ‐3′

### 4.11 RNA immunoprecipitation

The isolation of the total RNA was based on the experimental process described above. Five hundred nanograms of RNA was prepared for the RNA immunoprecipitation, and each RNA sample was combined with 5 μg of the anti‐N4‐acetylcytidine (ac4C) antibody (ab252215, Abcam). The RNA immunoprecipitation was performed using Pierce™ Protein A/G Magnetic Beads (88802, Thermo Fisher Scientific™) according to the manufacturer's instructions.

### 4.12 mRNA stability assay

We performed the actinomycin D assay to assess the stability of the mRNA. Cells cultured with the complete medium were used as the control group, and actinomycin D (a transcription inhibitor) was added to the culture medium to coculture with cells at a concentration of 200 nM for the experimental groups. After 0, 3, 6, 9 and 12 h of treatment with actinomycin D, the cells were collected for RNA extraction. Then, the total RNA was used to perform RT‐qPCR to detect the BCL9L, SOX4 and AKT1 levels at each time point, and the half‐life (*t*_1/2_) of mRNA was measured from the initial and final amounts of RNA.

$$K=\frac{\ln\left(C0\right)-\ln\left(C12\right)}{t12-t0};\;\;t_{1/2}=0.693/K.$$

### 4.13 Western blot analyses

The RIPA buffer (Invitrogen, USA) with protease and phosphatase inhibitors (Roche, Switzerland) was conducted to extract the total cell protein. The protein samples were loaded to sodium dodecyl sulphate‐polyacrylamide gel electrophoresis (SDS‐PAGE) and electrophoretically transferred to PVDF membranes (Millipore, Germany). The PVDF membranes were cut according to the predicted molecular weight and then blocked in 5% non‐fat milk for 1 h, followed by incubation with the NAT10 polyclonal antibody (1:1000; Abcam, Cambridge, UK), the BCL9L polyclonal antibody (1:1000; Affinity, Cat. AF7915), the SOX4 polyclonal antibody (1:1000; Affinity, Cat. DF2610), the AKT1 polyclonal antibody (1:1000; Affinity, Cat. AF0836) or the GAPDH monoclonal antibody (1:1000; Affinity, Cat. 2118S), respectively. The TBST‐diluted goat anti‐rabbit secondary antibody was applied and incubated at room temperature for 1 h. A Tanon 5200 Multiintelligent imaging system was used for the immunoblot analysis (Bio Tanon, China).

### 4.14 Polysome profile assay

The UMUC‐3 cells were cultured in 150 mm dishes and washed with ice‐cold 1× PBS. After removing the remaining PBS completely, 2 ml PBS (with 100 μg/ml CHX) was added. The cells were then scraped and collected in 15 ml tubes (2000×) to be centrifuged. Then, the PBS was removed, and 1.2 ml of a Polysome cell extraction buffer was added, incubated 10 min on ice and spun at 13 000 *g* for 10 min. We then collected the S/N, measured the OD and placed 1 ml of S/N on the top of 11 ml 10∼50% sucrose in a gradient tube. After centrifugation (36 000 rpm for 2–2.5 h at 4°C with max break), the samples were analysed by a Brandel Density Gradient Fractionation system.

### 4.15 Translation efficiency analysis

A puromycin assay was applied to evaluate the translation efficiency. Cells were incubated with puromycin (1 μM final concentration) for 30 min before being harvested. Then, we collected the protein as described above and loaded 15 μg of the protein onto an SDS polyacrylamide gel. The follow‐up procedures were the same as those for the Western blot analysis. The concentration of the first antibody was 1:1000.

### 4.16 Cell viability assay and flow cytometry analysis

The bladder cancer cells treated with shGFP or shNAT10 were cultured in 96‐well plates, whereas untreated cells were used as blank controls. Cell viability was evaluated and quantified by measuring the absorbance using a Tecan Infinite 200 PRO (Tecan, Switzerland) with the CCK‐8 assay. Seventy‐two hours after transient transfection, approximately 1 × 10^6^ cells were collected from 6‐well tissue culture plates. The proportion of apoptotic cells was determined with the Annexin V‐FITC Apoptosis Kit (eBioscience, USA) using a CytExpert flow cytometer (Beckman Coulter, Inc.).

### 4.17 Tumour cells sphere formation assay

The tumour cells were digested from the tissue culture plate, and the serum‐containing medium was discarded by adding PBS to wash the cells. Then, the cells were resuspended in a DMEM/F12 (1:1) medium containing 1 × B27, 20 ng/ml bFGF and 20 ng/ml EGF. These tumour cells were then plated on ultralow attachment plates at approximately 5000 cells per well and filled with 4 ml of a cancer stem cell culture medium. The cells were incubated at 37°C with 5% CO2, and the tumour spheres were observed and captured by inverted microscopy after approximately 10 days.

### 4.18 ALDEFLUOR assay

A total of 0.5–1 × 106 cells were prepared for the ALDEFLUOR assay and suspended in a ALDEFLUOR assay buffer. The cells were suspended within the ALDEFLUOR assay buffer containing an ALDH substrate. All the procedures were based on descriptions from the ALDEFLUOR™ ASSAY protocols. The stemness of cells was analysed with flow cytometry followed by the Aldehyde Dehydrogenase Based Cell Detection Kit (ALDEFLUOR™, 01700).

### 4.19 Cell migration and invasion assay

The migration and invasion assays were performed by suspending the cells in a medium lacking serum and plating 1 × 10^4^ cells into Boyden chambers (BD Bioscience, USA) with Matrigel (BD Bioscience, USA) 24 h after transfection. The lower chambers were incubated with 600 μl of the cell culture medium with 20% foetal bovine serum at 37°C for 48 h. Swabs were then used to remove the cells from the chambers. Paraformaldehyde was applied for fixation, and crystal violet was used to stain the cells that migrated or invaded outside their chamber. The stained cells were visualised by light microscopy, and images were captured using an SLR camera.

### 4.20 Statistical analysis

Two‐tailed unpaired *t*‐test was used to examine the differences between the two variables. The statistical significance of the three groups was measured by one‐way ANOVA. The difference in the Rip‐qPCR experiments in different groups was computed with two‐way ANOVA. The expression levels were expressed as dichotomous variables, and frequencies and percentages were computed for dichotomously independent variables. Cox proportional hazard regression was applied to examine the factors associated with death, and survival curves were obtained by the Kaplan–Meier method. A value of *p *< .05 was considered statistically significant. GraphPad Prism software (V. 7.04) was used for data management and statistical analyses.

## CONFLICT OF INTERESTS

The authors have declared that no conflict of interest exists.

## Supporting information

Supporting information.
**FIGURE S1**. The correlation of NAT10 with the gender and age. (A) The statistic of NAT10 IHC score in male and female patients. (B) IHC score of NAT10 in different ages from 42 to 85 years

Supporting information.
**FIGURE S2**. NAT10 could exert an influence on target genes expression in different stages. (A) The mRNA stability assay is adopted to detect the relative level of BCL9L after treatment with Actinomycin D for 0, 3, 6, 9 and 12 h. (B) The qPCR experiment is applied to examine the relative expression of SOX4. (C) The stability of AKT1 is measured by qPCR after treatment with Actinomycin D for 0, 3, 6, 9 and 12 h.

Supporting information.
**FIGURE S3**. High‐expressed BCL9L is significantly correlated with clinical parameters in patients with BLCA. (A) Representative images for IHC staining of BCL9L in para‐tumour or tumour samples. Scale bars: 50 μm. (B) IHC score is calculated through assessment of immunostaining in normal or tumour sections respectively (**p < .01). (C) Analysis of the correlation between BCL9L expression level and clinical features of BLCA patients (*p < .05, ***p < .001, ****p < .0001). (D) The survival curve is used to estimate the overall survival of 63 patients with low or high expression of BCL9L

Supporting information.
**FIGURE S4**. SOX4 shows significant prognostic value for patients with BLCA. (A) The expression pattern of SOX4 is shown in adjacent normal or cancer tissue. (B) IHC score is calculated according to the expression level of SOX4 in para‐tumour or tumour tissues (****p < .0001). (C) SOX4 is closely related to the clinical features, including tumour stage, lymph node metastasis, tumour grade and clinical stage (*p < .05, ***p < .001, ****p < .0001). (D) The overall survival of BLCA patients with low or high expression is analysed by Kaplan–Meier estimates

Supporting information.
**FIGURE S5**. Evaluation of the expression level of AKT1 and its correlation with clinical features in BLCA patients. (A) Representative images of immunostaining for AKT1 in corresponding normal or cancer tissue. (B) IHC score of AKT1 is based on immunostaining statistics in both adjacent or tumour tissues. (C) The correlation of AKT1 level with tumour stage, lymph node metastasis, tumour grade and clinical stage. (D) Kaplan–Meier method is applied to evaluate the survival time of BLCA patients expressed low or high AKT1

Supporting information.
**FIGURE S6**. Co‐expression analysis between NAT10 and its targets. (A) Analysis of NAT10 and BCL9L expression level in tissue microarray. (B) Co‐expression analysis of NAT10 and SOX4 is shown by the scatter plot. (C) The correlation of NAT10 with AKT1 is conducted using correlation analysis
