# Supplementary figures and images for: NAT10‐mediated mRNA N4‐acetylcytidine modification promotes bladder cancer progression
Source: Clin Transl Med. 2022 May 6;12(5):e738. doi: 10.1002/ctm2.738 (PMC9076013; doi:10.1002/ctm2.738)

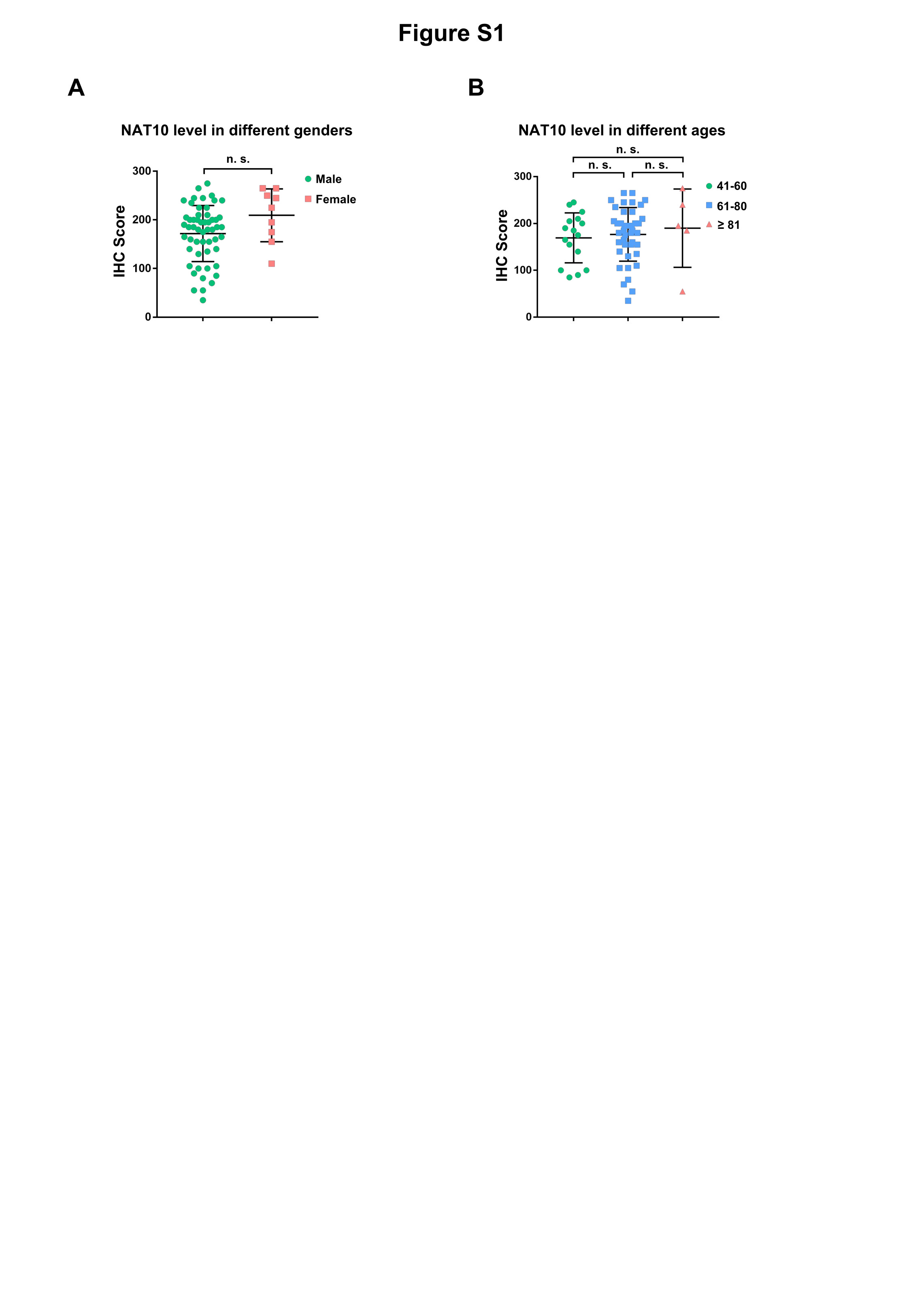

Supplement: Supplementary file 1 — Supporting information. FIGURE S1. The correlation of NAT10 with the gender and age. (A) The statistic of NAT10 IHC score in male and female patients. (B) IHC score of NAT10 in different ages from 42 to 85 years [file CTM2-12-e738-s006.tif]

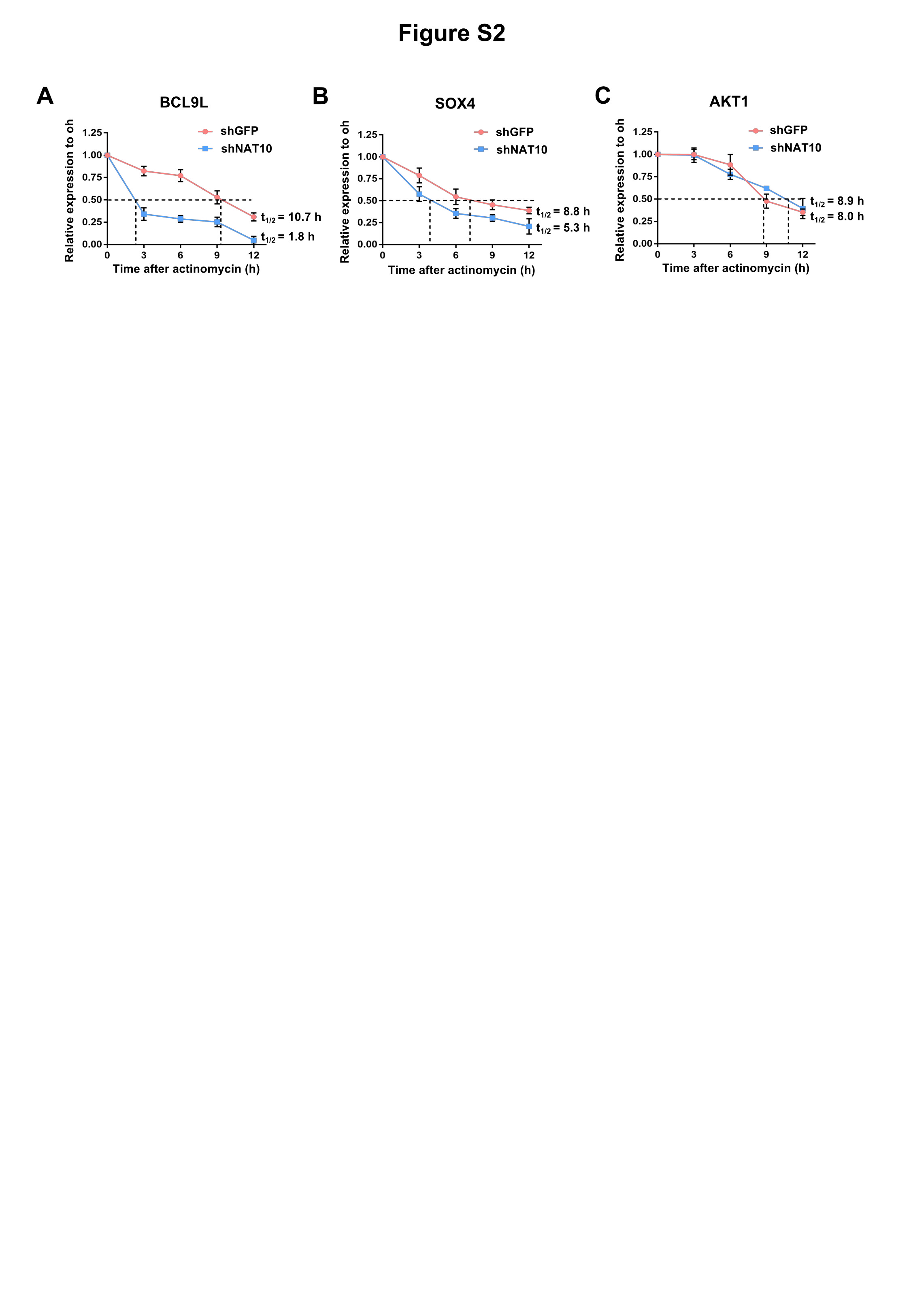

Supplement: Supplementary file 2 — Supporting information. FIGURE S2. NAT10 could exert an influence on target genes expression in different stages. (A) The mRNA stability assay is adopted to detect the relative level of BCL9L after treatment with Actinomycin D for 0, 3, 6, 9 and 12 h. (B) The qPCR experiment is applied to examine the relative expression of SOX4. (C) The stability of AKT1 is measured by qPCR after treatment with Actinomycin D for 0, 3, 6, 9 and 12 h. [file CTM2-12-e738-s001.tif]

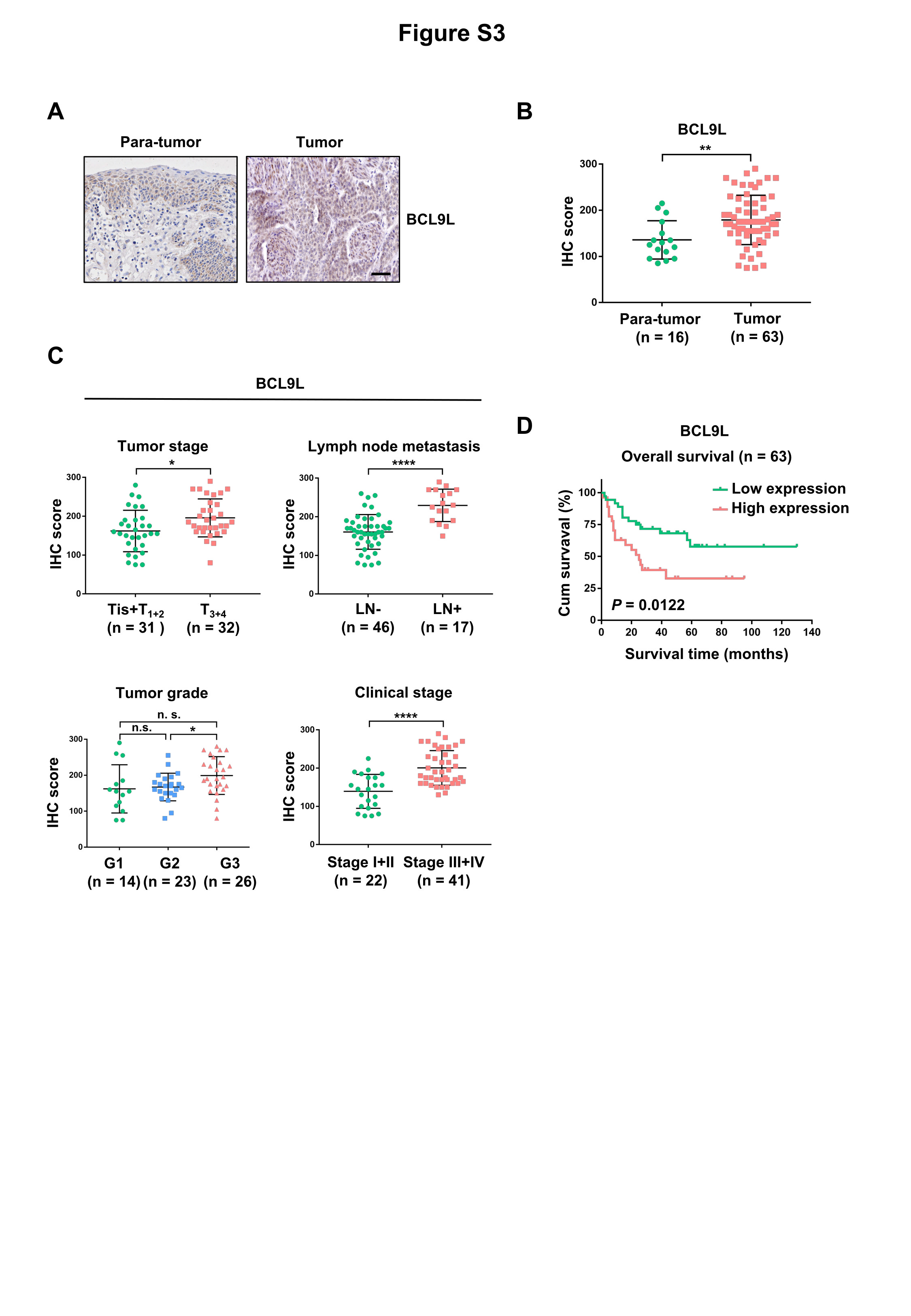

Supplement: Supplementary file 3 — Supporting information. FIGURE S3. High‐expressed BCL9L is significantly correlated with clinical parameters in patients with BLCA. (A) Representative images for IHC staining of BCL9L in para‐tumour or tumour samples. Scale bars: 50 μm. (B) IHC score is calculated through assessment of immunostaining in normal or tumour sections respectively (**p < .01). (C) Analysis of the correlation between BCL9L expression level and clinical features of BLCA patients (*p < .05, ***p < .001, ****p < .0001). (D) The survival curve is used to estimate the overall survival of 63 patients with low or high expression of BCL9L [file CTM2-12-e738-s004.tif]

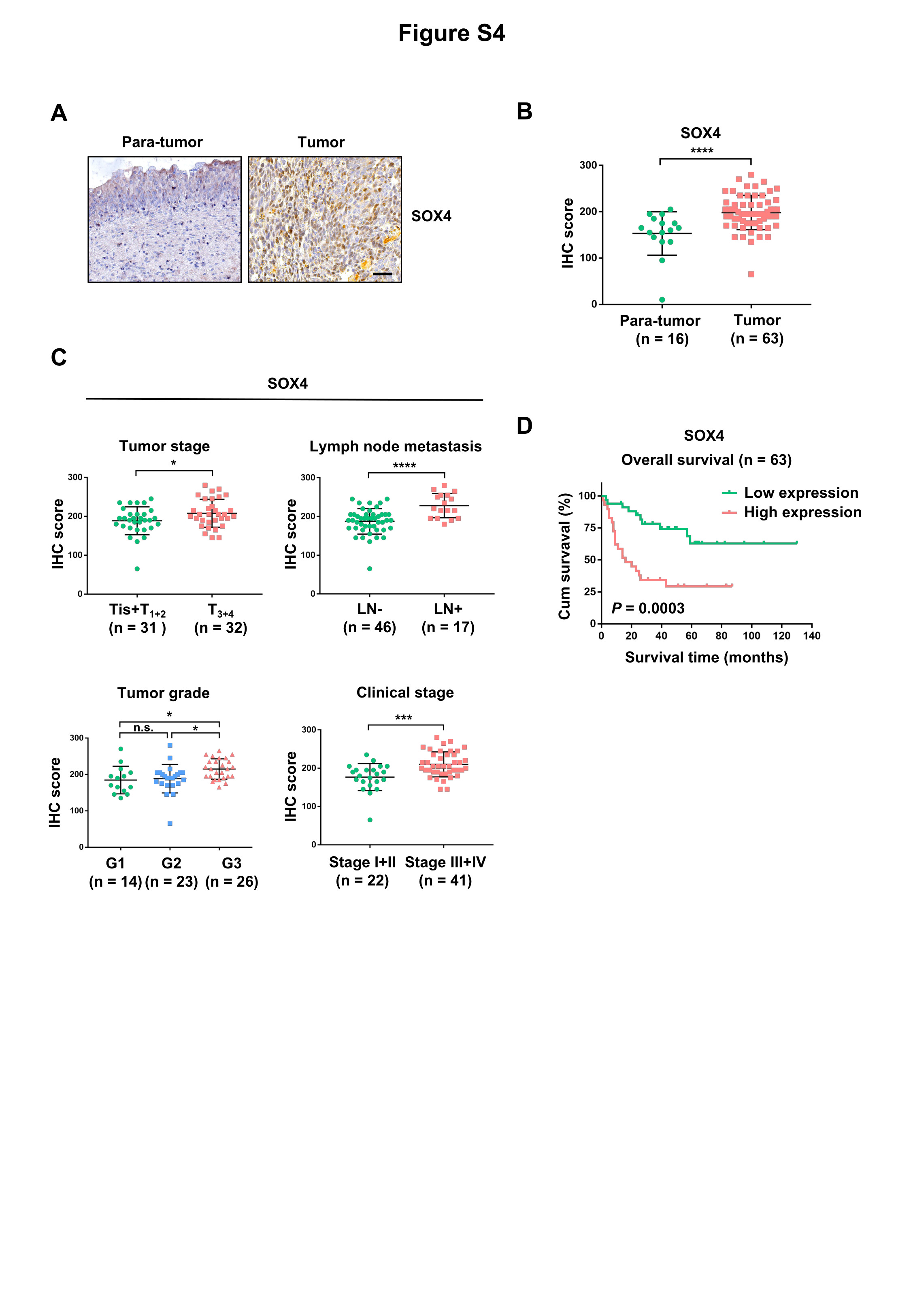

Supplement: Supplementary file 4 — Supporting information. FIGURE S4. SOX4 shows significant prognostic value for patients with BLCA. (A) The expression pattern of SOX4 is shown in adjacent normal or cancer tissue. (B) IHC score is calculated according to the expression level of SOX4 in para‐tumour or tumour tissues (****p < .0001). (C) SOX4 is closely related to the clinical features, including tumour stage, lymph node metastasis, tumour grade and clinical stage (*p < .05, ***p < .001, ****p < .0001). (D) The overall survival of BLCA patients with low or high expression is analysed by Kaplan–Meier estimates [file CTM2-12-e738-s005.tif]

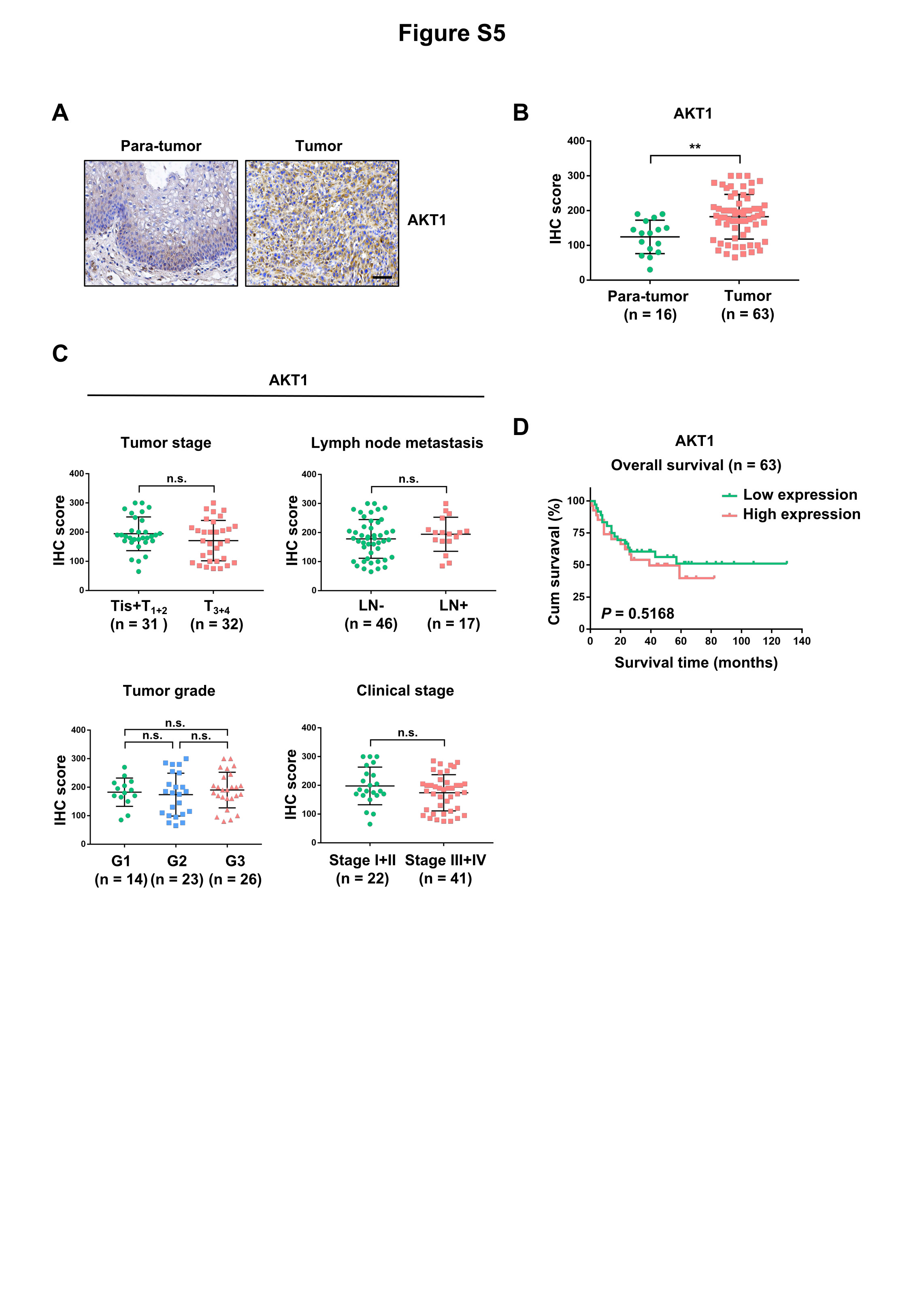

Supplement: Supplementary file 5 — Supporting information. FIGURE S5. Evaluation of the expression level of AKT1 and its correlation with clinical features in BLCA patients. (A) Representative images of immunostaining for AKT1 in corresponding normal or cancer tissue. (B) IHC score of AKT1 is based on immunostaining statistics in both adjacent or tumour tissues. (C) The correlation of AKT1 level with tumour stage, lymph node metastasis, tumour grade and clinical stage. (D) Kaplan–Meier method is applied to evaluate the survival time of BLCA patients expressed low or high AKT1 [file CTM2-12-e738-s002.tif]

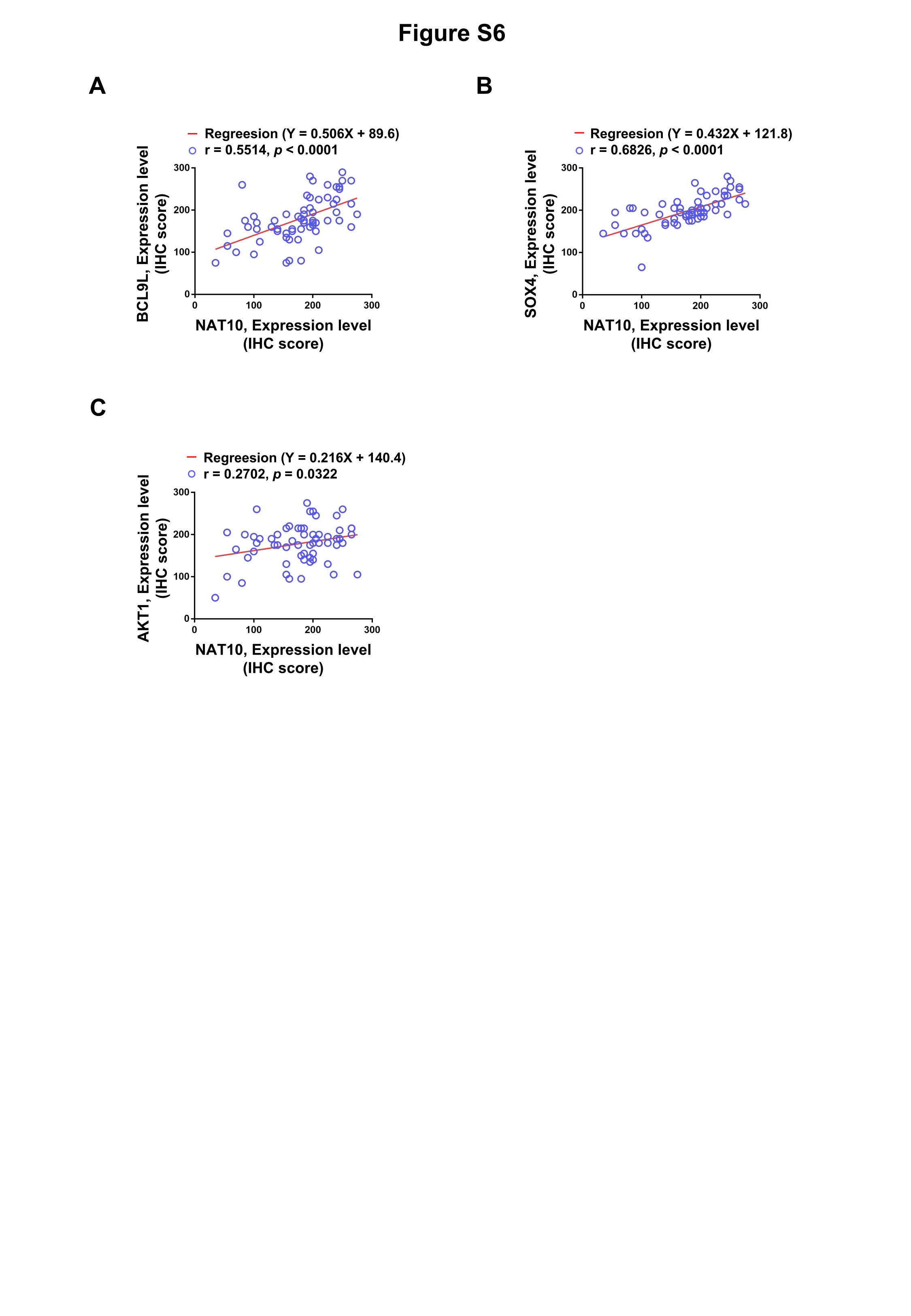

Supplement: Supplementary file 6 — Supporting information. FIGURE S6. Co‐expression analysis between NAT10 and its targets. (A) Analysis of NAT10 and BCL9L expression level in tissue microarray. (B) Co‐expression analysis of NAT10 and SOX4 is shown by the scatter plot. (C) The correlation of NAT10 with AKT1 is conducted using correlation analysis [file CTM2-12-e738-s003.tif]
